# Plasma Fatty Acid Profiling and Mathematical Estimation of the Omega-3 Index: Toward Diagnostic Tools in Atherosclerosis and Statin Therapy Monitoring

**DOI:** 10.3390/biomedicines13061383

**Published:** 2025-06-04

**Authors:** Nikolay Eroshchenko, Elena Danilova, Anastasiia Lomonosova, Alexey Antonik, Svetlana Lebedeva, Daria Gognieva, Dmitry Shchekochikhin, Tatiana Demura, Marina Krot, Nana Gogiberidze, Abram Syrkin, Philipp Kopylov

**Affiliations:** 1 Institute of Molecular Theranostics, Biomedical Science and Technology Park, I.M. Sechenov First Moscow State Medical University (Sechenov University), 119048 Moscow, Russia; phenolyat@gmail.com (E.D.); alexey.antonik@gmail.com (A.A.); 2 Department of Analytic Chemistry, Faculty of Chemistry, Lomonosov Moscow State University, 119991 Moscow, Russia; 3 World-Class Research Center «Digital Biodesign and Personalised Healthcare», Institute for Personalised Cardiology, I.M. Sechenov First Moscow State Medical University (Sechenov University), 119048 Moscow, Russia; lomonosova_a_a@staff.sechenov.ru (A.L.); gognieva_d_g@staff.sechenov.ru (D.G.); kopylov_f_yu@staff.sechenov.ru (P.K.); 4 Rehabilitation Room No. 2, University Clinical Hospital No. 1, I.M. Sechenov First Moscow State Medical University (Sechenov University), 119048 Moscow, Russia; 5 Department of Higher Geometry, Faculty of Mathematics and Mechanics, St. Petersburg State University, 7/9 Universitetskaya nab., 199034 St. Petersburg, Russia; 6 Department of Pharmacology, Institute of Pharmacy named after A.P. Nelyubin, I.M. Sechenov First Moscow State Medical University (Sechenov University), 119048 Moscow, Russia; lebedeva502@yandex.ru; 7 Department of Medical Elementology, Peoples’ Friendship University of Russia (RUDN University), 117198 Moscow, Russia; 8 Department of Cardiology, Functional and Ultrasound Diagnostics, N.V. Sklifosovsky Institute for Clinical Medicine, I.M. Sechenov First Moscow State Medical University (Sechenov University), 119048 Moscow, Russia; agishm@list.ru (D.S.); gogiberidze_n_a@staff.sechenov.ru (N.G.); syrkin_a_l@staff.sechenov.ru (A.S.); 9 City Clinical Hospital No. 1, 8 Leninsky Ave., 119049 Moscow, Russia; 10 Institute for Clinical Morphology and Digital Pathology, I.M. Sechenov First Moscow State Medical University (Sechenov University), 119048 Moscow, Russia; demura_t_a@staff.sechenov.ru (T.D.); krot_m_a@staff.sechenov.ru (M.K.)

**Keywords:** omega-3 fatty acids, carotid atherosclerosis, atherosclerotic plaques, statins, lipid metabolism, regression analysis

## Abstract

**Background/Objectives:** Omega-3 highly unsaturated fatty acids (HUFAs), particularly EPA and DHA, have anti-inflammatory and lipid-modulating properties for treating atherosclerosis. However, the relationship between plasma fatty acid profiles, omega-3 status, and statin efficacy in carotid atherosclerosis remains poorly defined. **Objectives:** This study evaluates plasma and plaque fatty acid (FA) composition, explores their associations with plaque stability, and examines the relationship of omega-3 levels, lipid biomarkers (VLDL-C, LDL-C, HDL-C, total cholesterol, and triglycerides) with statin and β-blocker treatment. A mathematical model was developed to predict the erythrocyte omega-3 index from plasma. **Methods:** In this case–control study, plasma and carotid plaques of 52 patients undergoing carotid endarterectomy were analyzed. Plasma was compared with that of 50 healthy controls. FAs were quantified by LC-MS/MS. Plaques were histologically classified as stable or unstable. **Results:** Atherosclerotic patients showed disturbed FA metabolism, including decreased plasma omega-3 EPA + DHA, SFAs and HUFAs, increased MUFAs, and impaired desaturase and elongase activity. Unstable plaques had higher MUFA and lower HUFA content compared with stable plaques. Significant correlations between plasma EPA + DHA and HDL-C and triglycerides were observed in statin-naïve patients, whereas statins appeared to attenuate these associations. Co-treatment with β-blockers had no significant effect. A validated logit-based model accurately predicted the erythrocyte omega-3 index from plasma (R^2^ = 0.782). **Conclusions:** Altered plasma and plaque FA profiles correlate with atherosclerosis’s plaque instability and inflammatory lipid profiles. Statins significantly influence these associations, suggesting their complex interaction with lipid metabolism. Plasma measurements of omega-3 fatty acids in combination with predictive modelling may be beneficial for diagnostic and therapeutic monitoring in carotid atherosclerosis.

## 1. Introduction

The progressive thickening and hardening of arterial walls due to atherosclerosis pose significant cardiovascular risks, especially when the carotid arteries are affected. Recent studies have focused on investigating the potential of eicosapentaenoic acid (EPA) and docosahexaenoic acid (DHA) omega-3 fatty acids in the treatment of atherosclerosis. These studies have investigated their capacity to mitigate inflammation and modify blood lipids, thereby presenting a promising avenue for therapeutic intervention. The omega-3 index, which is calculated by evaluating the ratios of eicosapentaenoic acid (EPA) and docosahexaenoic acid (DHA) relative to total membrane red blood cell fatty acids, is increasingly used as a biomarker of cardiovascular risk. Recent studies suggest that an omega-3 index of <3.43% predicts the progression of coronary plaques in statin-treated patients, while an omega-3 index of ≥4% prevents the progression of all coronary plaque subtypes [1]. This study raises the question of whether similar beneficial effects can be produced in carotid atherosclerosis.

Combination of statins with omega-3 fatty acids has been shown to have potential therapeutic benefits. However, practitioners continue to debate this combination and its effects on reducing LDL cholesterol. The medical research community has produced divergent results regarding the combined impact of these two treatments on reducing plaque progression and stabilising lesions [2]. Some studies have indicated minimal favourable outcomes, whilst others have demonstrated no additional benefit [3,4]. Research has indicated that low ratios of serum phospholipids omega-3/omega-6 are associated with elevated carotid plaque burden in patients with this condition [5].

The proposed synergy between omega-3 fatty acids and statins in reducing the progression of atherosclerosis is supported by their complementary effects on lipid metabolism, inflammation, and plaque stabilization. Statins primarily lower LDL cholesterol by inhibiting HMG-CoA reductase, while omega-3 fatty acids reduce triglycerides through enhanced clearance of very-low-density lipoprotein (VLDL) particles. This complementary action effectively addresses two lipid-related risk factors for atherosclerosis [6]. The anti-inflammatory effect has been observed both in statins and in omega-3 fatty acids. Omega-3 PUFAs improve endothelial function, promote vasodilatation through relaxation of smooth muscle cells and alter endothelium-dependent coronary vasodilation in heart transplant recipients [7]. They also ameliorate acute and chronic vascular inflammation [8]. Omega-3 fatty acids function as substrates for specialized pro-resolving mediators (SPMs), including resolvins and protectins. These specialized mediators are involved in regulating inflammation and stabilizing plaques. The effectiveness of these treatments is enhanced by statin-mediated modulation of inflammatory cytokines and decreased oxidative stress levels [2].

The primary cause of unstable plaque formation, i.e., oxidative stress, is diminished by utilizing these two agents. Reducing reactive oxygen species (ROS) production by omega-3 fatty acids works with statin-mediated LDL oxidation inhibition. These processes prevent lipid peroxidation-driven atherosclerosis development [9]. In addition to omega-3, other fatty acids, such as omega-6 and saturated fats, have been identified as contributing factors to the progression of atherosclerosis [5]. Research has indicated that excessive omega-6 consumption with low omega-3 intake results in elevated systemic inflammation and heightened vulnerability of atherosclerotic plaques [10]. The combination of dietary habits, genetic alterations in fatty acid desaturase enzymes, statin medication, and concomitant medical disorders (including diabetes and obesity) substantially shapes individual fatty acid concentrations and their effects on heart disease [11].

The combination of omega-3 fatty acids with statins shows great promise, yet the conclusive effectiveness regarding carotid atherosclerosis remains unknown, and data discrepancies require further research. The present study investigated a group of patients afflicted with carotid artery atherosclerosis. Blood plasma samples were collected, and surgical removal of the plaques was performed. The fatty acid profile in the blood plasma and atherosclerotic plaques was analyzed. Histological classification of the plaque types was based on their stability. In addition, the patient histories available included disease progression and details on medication use.

The present study investigated the fatty acid compositions in both plasma and atherosclerotic plaques, and explored potential relationships between fatty acid profiles and atherosclerosis, statin treatment, and the histological stability of carotid atherosclerotic plaques. Additionally, this study aimed to evaluate the correlation between omega-3 status and standard lipid parameter assessments of statin therapy, including measurements of very low-density lipoprotein cholesterol (VLDL-C), low-density lipoprotein cholesterol (LDL-C), high-density lipoprotein cholesterol (HDL-C), total cholesterol (TC), and triglycerides (TG) when available [12]. This study further explores the diagnostic potential of plasma fatty acid profiling in guiding therapeutic strategies for atherosclerosis.

This work is a development of the research previously conducted by Lomonosova et al. (2024) [13]. The present study was initially made within a registered clinical protocol focused on circulating biomarkers of plaque instability (ClinicalTrials.gov ID: NCT05680935), where blood plasma was selected as the primary biospecimen. The present fatty acid profiling assay was applied to gain a more comprehensive understanding of the lipid-related mechanisms involved in atherosclerosis. As red blood cell (RBC) fatty acid composition is widely regarded as the gold standard for determining the omega-3 index [14], and only plasma samples were available in this cohort, a mathematical model was developed to estimate RBC omega-3 levels from plasma values. This approach has been validated in previous studies and enables the assessment of long-term omega-3 status and its clinical relevance even when only plasma is available [15,16,17].

The present case–control study investigated advanced atherosclerosis by analyzing baseline plasma and plaque fatty acid profiles in patients under routine care without modifying existing treatment regimens or administering omega-3 supplementation.

## 2. Materials and Methods

### 2.1. Participants

The study followed the Declaration of Helsinki and was approved by the university’s ethics committee (Protocol Code No. 16-22, 1 September 2022), registered on ClinicalTrials.gov (NCT05680935). The patients provided written informed consent.

Blood plasma samples and atherosclerotic plaque tissue were collected from 52 patients with clinically confirmed atherosclerosis, at the Cardiology Clinic of the University Clinical Hospital No. 1 of Sechenov University (Moscow, Russia) from September 2022 to October 2023; these patients formed the Atherosclerosis group (AS).

Additionally, data from 69 random individuals (nursing and medical staff) were used, from whom blood plasma and erythrocyte mass samples had previously been collected (from January 2023 to August 2024). The dataset of these 69 individuals was utilized to construct a mathematical model that enabled the conversion of omega-3 fatty acid levels measured in plasma into estimated values for erythrocyte membranes (Supplementary File S1, Table S1).

Of these 69 participants, 50 apparently healthy individuals without diagnosed atherosclerosis were selected as the Control group.

Inclusion and non-inclusion criteria for each group are detailed below:

Inclusion criteria for the Atherosclerosis group were as follows: (1) age between 18 and 85 years; (2) provision of written informed consent; (3) confirmed diagnosis of hemodynamically significant stenosing atherosclerosis of the brachiocephalic arteries, verified by duplex ultrasound and/or multislice computed tomography (MSCT) angiography, including individuals with or without a history of transient ischemic attack (TIA) or stroke attributed to carotid artery disease; (4) availability of general blood analysis and lipid profile data; (5) outpatient follow-up or inpatient care at a research institution; and (6) patients undergoing carotid endarterectomy.

Non-inclusion criteria for the Atherosclerosis group were as follows: (1) chronic kidney disease stage 3b or higher (estimated glomerular filtration rate (eGFR) < 45 mL/min/1.73 m^2^); (2) presence of severe non-cardiovascular somatic diseases with a life expectancy of less than 6 months; (3) chronic diseases in an active exacerbation phase; (4) body weight less than 40 kg or greater than 125 kg; and (5) pregnancy.

Inclusion criteria for the Control group (individuals without signs of brachiocephalic artery atherosclerosis) were (1) age between 18 and 85 years; (2) provision of written informed consent; (3) absence of clinical symptoms suggestive of atherosclerosis; (4) absence of detectable brachiocephalic artery atherosclerosis based on duplex ultrasound and/or MSCT angiography; and (5) participation in outpatient follow-up or hospitalization at a research institution.

Non-inclusion criteria for the Control group were (1) a history or clinical evidence of systemic atherosclerosis or cardiovascular disease; (2) presence of autoimmune diseases, malignancies, chronic obstructive pulmonary disease, chronic kidney disease (stage ≥ 3) or liver cirrhosis; (3) acute infectious or inflammatory conditions at the time of sample collection; (4) exacerbation of chronic illnesses; (5) ongoing treatment with lipid-lowering, anti-inflammatory, or immunosuppressive medications; (6) body weight less than 40 kg or greater than 125 kg; and (7) pregnancy.

Non-exacerbated conditions such as diabetes mellitus, chronic gastritis, previous episodes of tonsillitis, seasonal allergic rhinitis, and well-controlled arterial hypertension (Stage 1, without cardiovascular complications or target organ damage) were not considered exclusionary in the control group.

Exclusion from the study for all participants was determined by withdrawal of informed consent at any stage of participation.

The median ages for the two groups (AS and Control) were 67 and 46 years, respectively. The atherosclerosis group demonstrated a higher prevalence of smoking, hypertension, diabetes, ischemic vascular issues, and kidney disease. In the first group, 76.9% (40 of 52) of patients with atherosclerosis were taking statins regularly, and there was also information on the use of ACE inhibitors, calcium antagonists, sartans, β-blockers, anticoagulants, and diuretics (Table 1).

### 2.2. Biological Samples

Blood samples (*n* = 121) were collected from the antecubital vein in tubes with anticoagulant K3EDTA after overnight fasting and processed through centrifugation at 1000× *g* for 10 min. Then, ¾ volume of the plasma was taken from above and transferred into a new empty tube following centrifugation at 2500× *g* for 15 min to sediment the platelets. The supernatant (blood plasma, *n* = 121) was aliquoted into 2.0 mL Eppendorf tubes. The erythrocytes (*n* = 69) were washed three times with a 0.9% sodium chloride solution, with the supernatant being removed each time. The blood fraction samples were stored at −80 °C.

Atherosclerotic plaques (*n* = 52) obtained during a planned carotid endarterectomy were divided into two parts. One-half of the sample was placed in a 10% neutral buffered formalin solution for histological analysis, while the other half was stored in tubes containing RNAprotect Tissue Reagent (QIAGEN, Venlo, The Netherlands) for subsequent LC-MS/MS analysis. The samples were then frozen and stored at −80 °C.

### 2.3. Histological Evaluation of Atherosclerotic Plaques

A histological analysis was performed at the Institute for clinical morphology and digital pathology (Sechenov University). Following excision, the plaques samples were fixed in 10% neutral buffered formalin, processed routinely, and embedded in paraffin. Serial 4–5 µm sections were cut and stained with hematoxylin and eosin (H&E) for histopathological examination. Microscopic evaluation was performed using a Leica DM2500 microscope (Leica Microsystems, Wetzlar, Germany) under magnifications of ×100, ×200, and ×400.

The study aimed to assess the morphological differences between stable and unstable atherosclerotic plaques, based on established histopathological features associated with plaque vulnerability [18]. A semi-quantitative scoring system was employed to evaluate plaque components: absence of a feature was scored as 0, low intensity as 1, moderate as 2, and high intensity as 3. The following histological characteristics were analyzed: lipid core size, fibrous cap thickness, inflammatory infiltrates, neoangiogenesis, calcification, surface ulceration, intraplaque hemorrhage, superimposed thrombosis. Representative histological images illustrating the morphological differences between stable and unstable plaques are presented in File S1, Figure S1.

Stable plaques were characterized by a small to moderately sized lipid core, a thick fibrous cap, and minimal or absent inflammation and neoangiogenesis. Secondary changes such as calcification, ulceration, hemorrhage, and thrombosis were either absent or present at low intensity.

In contrast, unstable plaques exhibited a large necrotic lipid core, thin or ruptured fibrous cap, and prominent inflammatory infiltration and neoangiogenesis, often accompanied by surface ulceration, intraplaque hemorrhage, and superimposed thrombosis—hallmarks of vulnerable plaques described in prior studies [18,19].

This comprehensive assessment enabled the classification of plaques as stable or unstable. Of the 52 plaques examined, 24 were deemed unstable (UP), while 28 were classified as stable (SP).

### 2.4. Reagents

Fatty acid methyl ester standards (Larodan, Solna, Sweden) included saturated (C12:0, C14:0, C16:0, C17:0, C18:0, C20:0, C22:0, C24:0), unsaturated omega-7 (C16:1n-7), omega-9 (C18:1n-9, C20:11n-9, C24:1n-9), *trans* fatty acids (C16:1n-7 *trans*, C18:1n-9 *trans*, C18:2n-6 *trans*), omega-6 (C18:2n-6, C18:3n-6, C20:2n-6, C20:3n-6, C20:4n-6, C22:4n-6), and omega-3 (EPA C20:5n-3, DPA C22:5n-3, DHA C22:6n-3).

The solvents used were methanol, acetonitrile, and isopropanol for LC-MS (Merck, Darmstadt, Germany). Deionized water was prepared with a Milli-Q Advantage A10 apparatus (Merck, Germany). Boron trifluoride–methanol solution (14% in methanol), 2,6-Di-*tert*-butyl-4-methylphenol (BHT, ≥99.0%, powder), formic acid (98%), and ammonium formate (for HPLC, ≥99.0%) were purchased from Sigma-Aldrich (Sigma-Aldrich, St. Louis, MO, USA).

### 2.5. Sample Preparation and HPLC-MS/MS Analysis

The plasma and erythrocyte samples were prepared and analyzed using the previously described method [20]. In summary, 40 μL of biological sample was mixed with 50 μL of methyl heptadecanoate internal standard solution in methanol (C17:0, 500 μg mL^−1^) and 910 μL of methanol with BHT 200 μg mL^−1^. The mixture was subjected to a 30-min shaking process, followed by a centrifugation step. Subsequently, 500 μL of the resultant mixture were transferred into a vial containing 500 μL of the boron trifluoride solution. Following a sealing and heating process at 100 °C for 90 min, the samples were cooled and then mixed with water at a 1:1 volume ratio. Subsequent analysis was conducted using HPLC-MS/MS.

Atherosclerotic plaque samples were thawed on ice. The tissue fragments were weighed and placed into Eppendorf tubes, which had been individually labelled. Each tube was loaded with one 5 mm stainless steel bead and four 1–2 mm diameter beads for homogenization. The Folch method was employed for lipid extraction, with 1 mL of chloroform: methanol (2:1, *v*/*v*) serving as the extraction solvent. The samples were then homogenized using a bead beater for three cycles at 7000 rpm, each lasting 20 s. Cooling intervals of approximately 5 min were implemented between each cycle to prevent overheating. The resulting extract was transferred into clean Eppendorf tubes and centrifuged at 13,000 rpm for 20 min at 10 °C. The superior portion was collected into new tubes. An aliquot of 40 µL of each sample was transferred into an Eppendorf tube and prepared analogous to the blood fraction described above.

The chromatographic separation was performed using the Shimadzu LC-20AD Liquid Chromatograph (Shimadzu, Kyoto, Japan) with a Phenomenex Kinetex C8 Column (2.6 µm, 3 mm × 100 mm). Eluent A comprised 0.1% formic acid in deionized water and 5 mM ammonium formate. Eluent B was composed of a mixture of acetonitrile and isopropanol (1:1) at a concentration of 99% and 1% deionized water, with 0.1% formic acid and 5 mM ammonium formate. FAME [NH_4_]^+^ adducts were detected on a Sciex 4500 QTRAP mass spectrometer (Sciex, Concord, ON, Canada) using electrospray ionization in positive mode.

### 2.6. Data Processing and Statistics

Targeted fatty acid profiling data were processed using Analyst 1.6.3 and MultiQuant 3.0 software (Sciex, Canada), GraphPad Prism 8.0.1 software (GraphPad Software, Boston, MA, USA), and R 4.3 statistical software and its packages. An a-priori power analysis (one-way ANOVA, effect size f = 0.40, α = 0.05, power = 0.80) performed with G*Power 3.1 indicated a minimum of 31 participants per group; therefore, 50 and 52 were enrolled in groups to accommodate potential dropouts. Sample values were considered unpaired and consistent, confirmed by the ROUT outlier test (Q = 1%). Normality was confirmed, and one-way ANOVA was performed. Statistical significance was set at a two-sided *p*-value of less than 0.05.

Descriptive statistics (mean, standard deviation, median, etc.) were calculated for quantitative variables and proportions for categorical data. Welch’s *t*-test and the Mann–Whitney U test were used for normally and non-normally distributed data, respectively. Pearson’s chi-squared test was used for categorical data, and Fisher’s exact test was used when necessary. Linear regression for continuous data was used to assess bivariate relationships between different variables within the dataset. The Shapiro–Wilk test was used to evaluate the normality of the residuals in the linear model and the Q-Q plot. A Bland–Altman plot was used to compare the reported and predicted results.

## 3. Results

### 3.1. Fatty Acid Profile of Blood Plasma in Atherosclerosis

The blood plasma from the control group and patients with atherosclerosis were analyzed and compared fatty acid content. We also studied the ratio of precursors to product fatty acids in the metabolic pathways of saturated, monounsaturated, omega-6 and -3 fatty acids to assess the activity of enzyme systems. Comparative analysis of plasma fatty acid composition revealed several statistically significant differences between healthy controls (*n* = 50) and patients with atherosclerosis (AS, *n* = 52).

Saturated fatty acids (SFA) were significantly lower in AS patients (34.04 ± 1.38%) compared with controls (37.61 ± 3.88%, *p* < 0.0001). In contrast, total unsaturated fatty acids (UFA) were correspondingly elevated in the AS group (65.96 ± 1.38%) compared to controls (62.40 ± 3.88%, *p* < 0.0001). Among the UFAs, monounsaturated fatty acids (MUFA) were significantly higher in AS patients (30.94 ± 3.50%) compared to controls (26.15 ± 3.64%, *p* < 0.0001), whereas polyunsaturated fatty acids (PUFA) were not significantly different (*p* = 0.2066).

**Figure 1:**
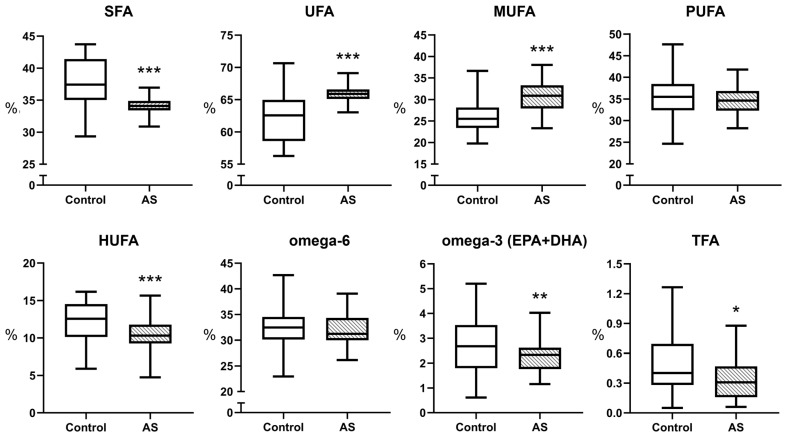
Comparative plasma levels of major fatty acid classes in healthy individuals (Control) and atherosclerosis patients (AS). The asterisks indicate that there are statistically significant differences versus the control group *p*-value < 0.05 (*), < 0.01 (**), and < 0.001 (***). All values in the figure are expressed as percentages (%). The boxplots of parameters TFA and HUFA indicate the median and [IQR 25–75%]. The lines inside the boxplots of other parameters indicate the mean ± SD.

Highly unsaturated fatty acids (HUFA) were reduced in AS (10.31 [9.25–11.78]) compared with controls (12.55 [10.12–14.52], *p* = 0.0009). In particular, omega-3 EPA + DHA levels were significantly lower in AS (2.23 ± 0.58%) compared with controls (2.70 ± 1.07%, *p* = 0.0068), with a corresponding reduction in EPA (0.42 [0.30–0.56] vs. 0.56 [0.34–0.79], *p* = 0.0159). Omega-6 PUFAs remained statistically similar between groups. The *trans* fatty acid (TFA) index was lower in AS (0.31 [0.16–0.47]) than in controls (0.40 [0.28–0.69], *p* < 0.0001) (Figure 1).

The lower TFA index observed in AS patients seems contrary to expectations, as *trans* fats have historically been associated with cardiovascular risk. The most likely reason for these observations is due to dietary changes and lifestyle interventions after diagnosis rather than endogenous metabolic changes alone [21].

**Figure 2:**
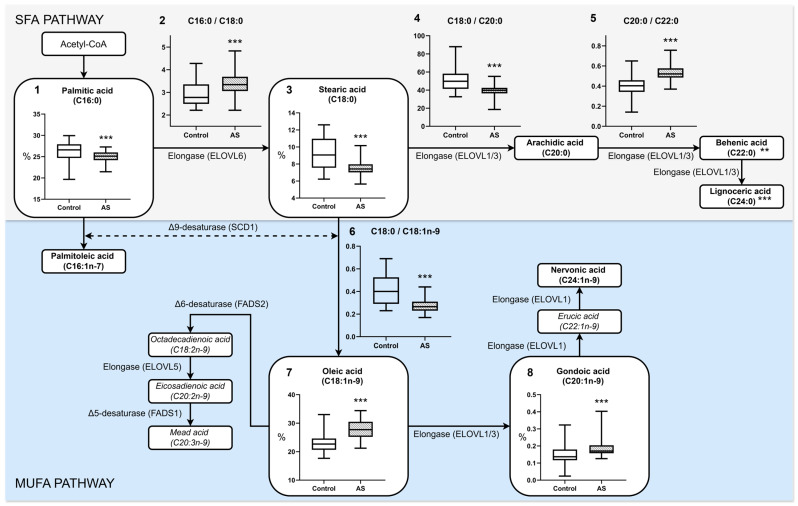
Biosynthesis pathway of SFA and MUFA with graph data of fatty acid composition in the plasma of the control and atherosclerosis (AS) group. 1—Palmitic acid (C16:0) % content; 2—C16:0/C18:0 ratio; 3—Stearic acid (C18:0) % content; 4—C18:0/20:0 ratio; 5—C20:0/22:0 ratio; 6—C18:0/C18:1n-9 ratio; 7—Oleic acid (C18:1n-9) % content; 8—Gondoic acid (C20:1n-9) % content. Statistically significant differences are indicated by ** (*p* < 0.01), and *** (*p* < 0.001), which were evaluated by an appropriate test. The enzyme nomenclature and metabolic scheme presented are based on previously published data [24,25,26,27,28,29]. FAs determined by the applied HPLC-MS/MS method are highlighted in bold. The other FAs involved in metabolism are written in italics. Fatty acid contents are expressed as percentages of the total identified fatty acids (%), whereas all fatty acid ratios (e.g., C16:0/C18:0) are reported as dimensionless numbers. The boxplots of parameters for stearic acid (C18:0) and oleic acid (C18:1n-9) indicate the mean ± SD. The other parameters’ boxplots are the median and [IQR 25–75%].

A detailed pathway diagram comparing the fatty acid composition in the plasma of healthy subjects (control) and patients with atherosclerosis (AS), focusing on the biosynthesis of saturated fatty acids (SFA) and monounsaturated fatty acids (MUFA), is shown in Figure 2. Fatty acid levels and FA ratios with statistically significant *p*-values are presented in Table 2. Information on all detected FA and ratios is presented in File S1, Table S2.

Decreased plasma levels of palmitic acid (C16:0), stearic acid (C18:0), behenic acid (C22:0), and lignoceric acid (C24:0) in AS patients suggest impaired elongation of SFAs (*p* values ranging from <0.0001 to 0.0009). Altered ratios, such as an increased C16:0/C18:0 ratio (*p* < 0.0001), indicate reduced palmitate to stearate elongation, implying reduced ELOVL6 activity [22]. Decreased C18:0/C20:0 indicates reduced capacity for elongation from stearate to arachidic acid. Increased C20:0/C22:0 and C22:0/C24:0 ratios indicate a stepwise disruption of very long chain fatty acid (VLCFA) elongation, likely involving ELOVL1 and ELOVL3 [23].

The AS group had higher total MUFA levels, which researchers have linked to increased desaturation activity. Significant increases in oleic acid (C18:1n-9) and gondoic acid (C20:1n-9) are observed in AS patients (*p* < 0.0001 for both). The C18:0/C18:1n-9 ratio is significantly decreased in AS patients (0.265 vs. 0.400, *p* < 0.0001), indicating upregulated SCD1 activity. SCD1 catalyzes the desaturation of stearic acid (C18:0) to oleic acid (C18:1n-9) [24].

**Figure 3:**
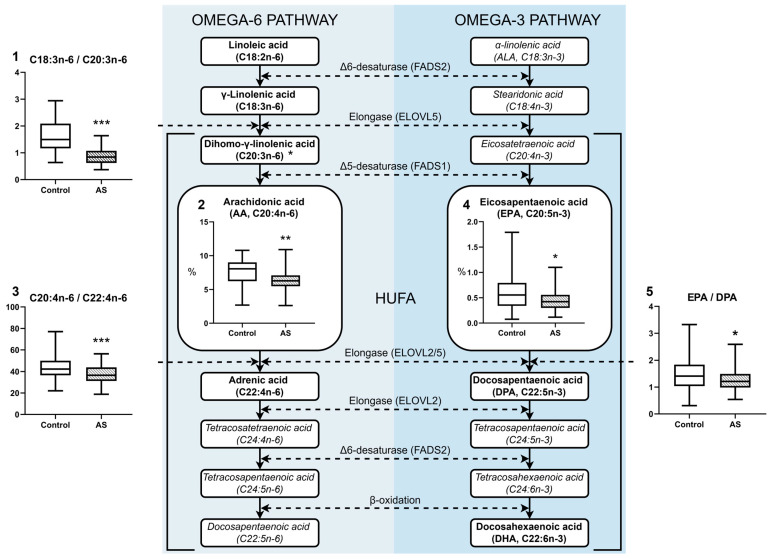
Biosynthetic pathway of omega-3 and omega-6 with graphical data of fatty acid composition in plasma of control and atherosclerosis (AS) group. 1—C18:3n-6/C20:3n-6 ratio; 2—arachidonic acid (C20:4n-6) % content; 3—C20:4n-6/C22:4n-6 ratio; 4—EPA % content; 5—EPA/DPA ratio. Statistically significant differences are indicated by * (*p* < 0.05), ** (*p* < 0.01) and *** (*p* < 0.001), which were evaluated by an appropriate test. Enzyme names and the metabolic pathway diagram are adapted from literature sources as cited [34,35]. FAs determined by the applied HPLC-MS/MS method are highlighted in bold. The other FAs involved in metabolism are written in italics. Fatty acid contents are expressed as percentages of the total identified fatty acids (%), whereas all fatty acid ratios (e.g., EPA/DHA) are reported as dimensionless numbers. All the boxplots indicate the median and [IQR 25–75%].

The omega-3 and omega-6 polyunsaturated fatty acid metabolism pathways were further analysed similarly (Figure 3). Patients with atherosclerosis showed significantly reduced levels of omega-6 C20:3n-6, together with C20:4n-6 (*p* = 0.0303 and *p* = 0.0033) fatty acids, in addition to reduced omega-3 EPA (*p* = 0.0159), while showing reduced total omega-3 levels and altered metabolic conversion ratios. Lower levels of C20:3n-6 and C20:4n-6 suggest reduced activity of Δ5-desaturase (FADS1), which converts dihomo-γ-linolenic acid (C20:3n-6) to arachidonic acid (C20:4n-6) [30,31]. The observations of reduced EPA and total omega-3 levels and a reduced EPA/DPA ratio suggest impaired omega-3 PUFA elongation involving ELOVL2 and ELOVL5 elongases [23]. In patients with AS, the ratio of C18:3n-6 to C20:3n-6 decreased significantly. However, C18:2n-6 and C18:3n-6 remained stable, demonstrating impaired elongation of γ-linolenic acid (C18:3n-6) to dihomo-γ-linolenic acid (C20:3n-6), possibly due to reduced activity of the elongase ELOVL5 rather than reduced Δ6-desaturase (FADS2) function [32]. However, there is a trend towards a decrease in the mean values of acids from C18:3n-6 in the AS group, although this is not statistically significant. The ratio of C18:2-n6/C20:4n-6 is significantly lower in the AS group (*p* = 0.0054), which indirectly indicates a decrease in delta-6-desaturase (FADS2) activity [31]. Lower HUFA levels combined with altered omega-6 ratios (e.g., C20:4n-6/C22:4n-6) indicate that lipid elongation and desaturation processes were significantly affected [33].

### 3.2. Fatty Acid Profile of Atherosclerotic Plaques

Fatty acid profiling of atherosclerotic plaques histologically classified as stable (*n* = 28) or unstable (*n* = 24) revealed several statistically significant differences. Although total saturated fatty acids (SFA) and unsaturated fatty acids (UFA) did not differ significantly (*p* = 0.1473), UFA were elevated in unstable plaques (68.34 ± 5.16%) compared with stable plaques (66.03 ± 6.40%) (Table 2). Information on all detected FA and ratios is presented in the File S1, Table S3.

Among the unsaturated fatty acid classes, monounsaturated fatty acids (MUFA) were significantly increased in unstable plaques (33.62 ± 5.11%) compared with stable plaques (30.90 ± 4.15%, *p* = 0.0323). Polyunsaturated fatty acids (PUFA) remained comparable between groups (*p* = 0.5543), but highly unsaturated fatty acids (HUFA), a subset reflecting ≥20-carbon PUFAs, were significantly reduced in unstable plaques (10.68 ± 1.74%) compared with stable plaques (11.91 ± 1.79%, *p* = 0.0129).

Omega-3 fatty acids EPA and DPA were reduced in unstable lesions (1.76 [1.35–1.89]%) compared with stable lesions (1.90 [1.54–2.48]%, *p* = 0.0389), mainly due to a reduction in DHA (1.11 [0.96–1.35]% vs. 1.29 [1.16–1.62]%, *p* = 0.0247). EPA levels tended to be lower in unstable plaques (0.48 [0.34–0.62]%) than in stable plaques (0.57 [0.37–0.78]%), but the difference was not statistically significant (*p* = 0.1318). Notably, DPA levels were significantly lower in unstable lesions (0.26 [0.23–0.36]% vs. 0.38 [0.27–0.47]%, *p* = 0.0335). The same effect was not observed in plasma. The major omega-6 fatty acid arachidonic acid (C20:4n-6) was significantly reduced in unstable plaques (6.17 ± 1.23%) compared with stable plaques (7.02 ± 0.97%, *p* = 0.0061), while total omega-6 levels were unchanged (*p* = 0.6559).

The MUFA components oleic acid (C18:1n-9) and gondoic acid (C20:1n-9) were both significantly increased in unstable plaques (*p* = 0.0217 and *p* = 0.0407, respectively). The omega-7 MUFA palmitoleic acid (C16:1n-7) was also higher in unstable plaques (3.02 [2.56–3.79]%) compared with stable plaques (2.79 [2.16–3.34]%, *p* = 0.0361). In the saturated fatty acid subgroup, very long chain saturated fatty acids (VLC-SFAs), such as behenic acid (C22:0) and lignoceric acid (C24:0), were significantly lower in unstable lesions (*p* = 0.0238 and *p* = 0.0410). At the same time, C20:0 also decreased significantly (*p* = 0.0303).

### 3.3. Effect of Statin Therapy on Plasma Fatty Acid Composition

**Figure 4:**
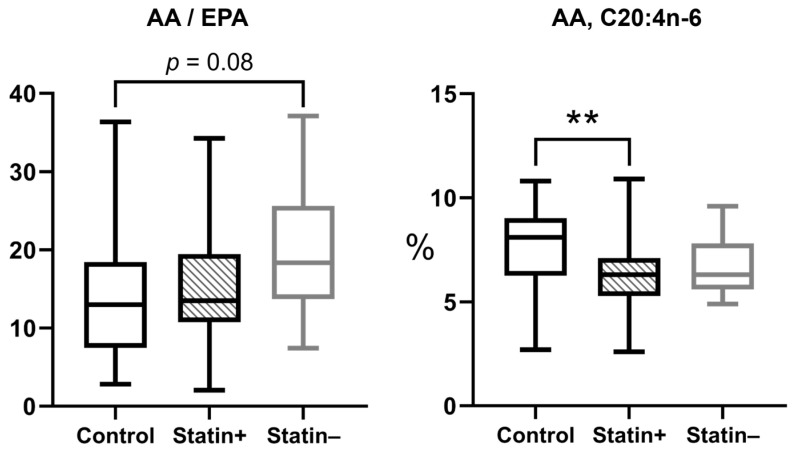
Differences in fatty acid content in the plasma of control and atherosclerosis group with (Statin+) or without statin therapy (Statin−). Statistically significant differences are indicated by ** (*p* < 0.01), evaluated by the Kruskal–Wallis test. AA content is expressed as a percentage of the total identified fatty acids (%), whereas AA/APE ratio is reported as dimensionless numbers. All the boxplots indicate the median and [IQR 25–75%].

In a previous study, statistical analysis showed no significant difference in plasma fatty acid levels between patients with stable and unstable atherosclerotic plaques [13]. This study investigated the possible correlation between fatty acid composition and statin therapy. Within the group of patients with atherosclerosis, there was generally no difference in plasma fatty acid composition with (Statin+) or without (Statin−) statin therapy. However, some differences were found when comparing arachidonic acid and EPA. The difference in the AA/EPA ratio was greater in the control vs. statin− group (*p* = 0.08), whereas the Control vs. Statin+ groups did not differ significantly (*p* > 0.99). The difference in AA% content was statistically significant in the Control vs. Statin+ group (*p* = 0.0064), while the Control vs. Statin− groups did not differ (*p* = 0.36) (Figure 4).

### 3.4. Linear and Logit Regression Models for Translating Plasma to Erythrocyte Omega-3 Index

Following previous studies by Stark et al. (2016) and Hu et al. (2017), we studied the bivariate relationship between omega-3 EPA + DHA in plasma and erythrocyte blood fractions [16,17]. We applied our developed method to analyze plasma and erythrocyte samples from 69 individuals, and the sample information is presented in File S1, Table S1. Our mathematical model was constructed and validated using paired plasma and erythrocyte samples from a representative cohort of the Russian population. The analytical LC-MS/MS method is based on analysing total lipids in plasma and erythrocytes [20].

**Figure 5:**
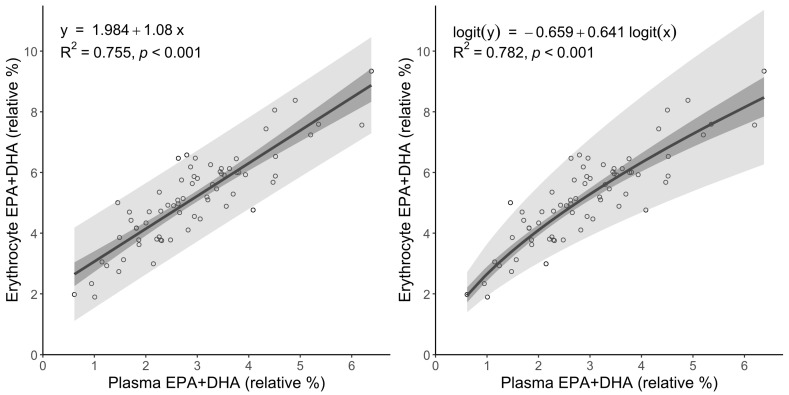
Linear regression (**left**) and logit-transformed regression (**right**) of EPA + DHA (%) between plasma and erythrocyte fractions (total lipids fraction). The logit transformation is defined as logit(y) = log (y/(100% − y)). The light gray area represents 95% prediction bands; the dark gray area represents 95% confidence bands (α = 0.05).

Our study showed a strong linear correlation between plasma and erythrocyte EPA + DHA levels (Figure 5, left), with a coefficient of determination of approximately R^2^ = 0.75 (*p* < 0.001). This suggests that erythrocyte omega-3 levels can be reasonably estimated from plasma using a simple linear model, which is valuable in settings where erythrocyte measurements are unavailable.

The predictive accuracy was enhanced by performing a logit transformation on the plasma data in conjunction with the erythrocyte data, given that the omega-3 index functions within the constrained interval (0.1). Linear regression analysis on transformed variables yielded enhanced outcomes, evidenced by an R^2^ value of 0.782 (Figure 5, right) and residuals that demonstrated improved conformance to normality. This assessment was facilitated through Q–Q plots and the Shapiro–Wilk test (*p* = 0.97 vs. 0.76 for visual data check File S1, Figure S2). The logit-transformed model demonstrated improved statistical performance; however, the transformed erythrocyte data did not strictly fulfil the normality requirements, necessitating careful interpretation of prediction intervals. The Bland-Altman statistics were typically characterized by equal agreement between both methods and the reported data. The second method demonstrated slight bias (0.046% vs. 0% for the linear model), though the spread was marginally lower (2.83% vs. 2.9% for linear).

A comparative assessment was conducted to evaluate the predictive power of the mathematical predictions for converting plasma total lipid measurements to erythrocyte omega-3 index values. The obtained mathematical predictions were assessed using the logit-based regression model developed in this study, compared with the meta-regression model proposed by Hu et al. (2017) [17]. Compared with the erythrocyte omega-3 index measured directly, the logit-transformed model attained an R^2^ of 0.782 (Figure 5, right), signifying a substantial degree of explanatory power and predictive precision. In contrast, the Hu et al. model yielded an R^2^ of 0.722, highlighting slightly lower model performance on the same dataset (File S1, Figure S3).

The plot demonstrates a high correlation coefficient (R^2^ = 0.977) between the two models, proving that our approach is adequate for measurements and calculations (File S1, Figure S3). The findings indicate that the logit-based regression model is optimal for translating the plasma omega-3 index to the erythrocyte. Residual distribution analysis demonstrates that the logit model results in smaller and more homoscedastic residuals, indicating reduced systematic bias and enhanced consistency in accuracy across the entire range of omega-3 index values. Bland–Altman analysis confirmed equal agreement between all three methods (linear model, logit transformation and Hu et al. (2017) model [17]) and reported data (File S1, Table S4). The second method demonstrated slight bias (0.05% vs. 0% for the linear model), though the spread was marginally lower (2.83% vs. 2.9% for the linear model). The third model provided a higher spread and bias (File S1, Figure S4).

Utilizing logit-transformed regression, the erythrocyte omega-3 index (EPA + DHA) was estimated in patients with atherosclerosis, with the estimation being based on their plasma EPA + DHA values. The plasma EPA + DHA concentration was measured at 2.23% (±0.58%), and the predicted erythrocyte omega-3 index was 4.35% (±0.72%), ranging from 2.90% to 6.37%. Notably, 18 subjects (34.6%) exhibited estimated omega-3 indices below 4%, which is regarded as the lower threshold for cardiovascular protection [36]. This finding underscores a notable proportion of patients who may exhibit suboptimal omega-3 status.

### 3.5. Association Between Statin Efficacy and Omega-3 PUFAs

The correlation between omega-3 fatty acid levels and measures of lipid metabolism was also studied, including very-low-density lipoprotein cholesterol (VLDL-C), low-density lipoprotein cholesterol (LDL-C), high-density lipoprotein cholesterol (HDL-C), total cholesterol (TC), and triglycerides (TG) in statin-treated (Statin+, *n* = 40) and statin-naive (Statin−, *n* = 12) patients. For visual data, see File S1, Figure S5.

*VLDL-C*. In patients treated with statins, omega-3 EPA + DHA levels demonstrated a mild and non-significant inverse correlation with VLDL-C (R^2^ = 0.024, *p* = 0.357). The predominant function of statins in the inhibition of hepatic cholesterol and lipoprotein synthesis may provide a potential explanation for this observation. The investigation of Statin- patients revealed an inverse correlation between omega-3 FA and VLDL (R^2^ = 0.485, *p* = 0.055), although this association was not statistically significant. However, this logically connects with proven mechanisms by which omega-3 FA reduce VLDL, involving decreased hepatic lipogenesis and increased β-oxidation [37].

*LDL-C*. No significant correlation was observed between omega-3 and LDL-C in the Statin+ group (R^2^ < 0.001, *p* = 0.989), which is likely to have been due to the dominant LDL-lowering mechanism of statins through HMG-CoA reductase inhibition. In Statin–, the relationship between statin data and omega-3 FA exhibited an inverse pattern that was not statistically significant (R^2^ = 0.271, *p* = 0.123). This finding suggests a potential, albeit limited, impact on LDL particle size and hepatic clearance processes [38,39].

*HDL-C*. In the Statin+ group, omega-3 levels demonstrated a weak and non-significant correlation with HDL-C (R^2^ = 0.035, *p* = 0.265). In patients in the Statin- group, a robust and statistically significant positive correlation was observed between omega-3 and HDL levels (R^2^ = 0.626, *p* = 0.011). This fact suggests that the eicosapentaenoic acid (EPA) and docosahexaenoic acid (DHA) components present may enhance the functionality of HDL by increasing cholesterol efflux and apolipoprotein A-I expression [40].

*Total Cholesterol (TC)*. No correlation was observed in Statin+ patients between omega-3s and TC (R^2^ = 0.0004, *p* = 0.902), indicating cholesterol levels are predominantly statin-dependent. The results from the Statin− group showed a moderate negative pattern (R^2^ = 0.322, *p* = 0.069), which indicated omega-3 FA might lower TC levels through their effects on lipogenesis and cholesterol clearance [41].

*Triglycerides (TG)*. Results indicating non-significant negative correlation were observed between omega-3 FA and triglycerides in the Statin+ group (R^2^ = 0.082, *p* = 0.077). The present study found that the statistical association became moderate and significant in the Statin– group, thereby demonstrating the potential of eicosapentaenoic acid (EPA) and docosahexaenoic acid (DHA) to decrease triglycerides (R^2^ = 0.453, *p* = 0.047) [41].

### 3.6. Concomitant Use of Statins and β-Blockers

A comprehensive review of the medications ingested by patients undergoing statin therapy was conducted during the medical history interview (*n* = 40). The following pharmaceuticals have been identified as having a proven effect on lipid metabolism: beta-blockers (*n* = 25), thiazide diuretics (hydrochlorothiazide) (*n* = 3), loop diuretics (torasemide) (*n* = 2), thiazide-like diuretics (indapamide) (*n* = 6), and xanthine oxidase inhibitors (allopurinol) (*n* = 5). Among the list, as mentioned earlier, β-blockers were used by a significant number of patients in conjunction with statins; therefore, they were chosen for an additional study of the efficacy of statin therapy. The correlation between omega-3 fatty acid levels and lipid metabolism assays was investigated considering β-blockers intake, forming statin + β-blocker (Statin + BB, *n* = 25) and statin-only (Statin only, *n* = 15) groups. For visual data check File S1, Figure S6.

*VLDL-C*. The relationship between omega-3 and VLDL-C was insignificant in the Statin + BB group (R^2^ = 0.006, *p* = 0.736), indicating that β-blockers do not affect statin-mediated lipid changes. The relationship observed in the statin-only group exhibited an inverse trend that did not reach statistical significance (R^2^ = 0.105, *p* = 0.240). This finding suggests that omega-3 fatty acids may have a modest effect on reducing VLDL-C levels.

*LDL-C*. The combination of statin and β-blocker therapy overshadows any omega-3 effects on LDL-C because no significant relationship emerged from the analysis (R^2^ = 0.028, *p* = 0.443). The statin-only group exhibited an inverse trend that was not statistically significant (R^2^ = 0.137, *p* = 0.174), indicative of the limited impact of omega-3 fatty acids on LDL-C reduction. This may be attributed to hepatic LDL receptor activity or particle remodelling alterations. However, the statistical power is insufficient to ascertain this effect [41].

*HDL-C*. No significant association was observed between omega-3 and HDL-C levels in the Statin + BB group (R^2^ = 0.045, *p* = 0.341). This observation suggests that omega-3 does not exert a significant influence on the regulation of HDL-C during combined therapy. The statin-only group displayed a modest, non-significant positive association (R^2^ = 0.018, *p* = 0.635), suggesting that omega-3 fatty acids exerted no notable independent influence on HDL-C levels.

*Total Cholesterol (TC)*. The Statin + BB group demonstrated no significant relationship between omega-3 and total cholesterol levels (R^2^ < 0.002, *p* = 0.869), indicating that omega-3 exerts no substantial effect on overall cholesterol levels while patients receive this combined treatment. The statin-only group revealed an inverse trend that did not reach statistical significance (R^2^ = 0.117, *p* = 0.211).

*Triglycerides (TG)*. The omega-3 levels failed to correlate significantly with triglyceride (TG) levels in the Statin + BB group (R^2^ = 0.069, *p* = 0.217). The statin-only group exhibited an inverse correlation with triglycerides, which was more pronounced, albeit not statistically significant (R^2^ = 0.188, *p* = 0.107). This finding indicates a potential for omega-3 FA to reduce triglycerides in conjunction with statins synergistically [41].

## 4. Discussion

The study found that patients with advanced carotid atherosclerosis exhibited significant alterations in their fatty acid profiles in both blood plasma and carotid plaques. These patients demonstrate persistent metabolic disruption to lipid processing, modifying desaturation and elongation events in saturated (SFA), monounsaturated (MUFA) and polyunsaturated (PUFA) fatty acids, including omega-3 (EPA + DHA) and omega-6. These alterations indicate complex modifications to metabolic enzyme activities, engendering pro-inflammatory and lipotoxic fatty acid conditions that promote atherosclerosis development.

The findings demonstrate a reduction in stearic acid (C18:0) and an increase in oleic acid (C18:1n-9), accompanied by a decrease in the C18:0/C18:1n-9 ratio. This result is consistent with the hypothesis that stearoyl-CoA desaturase-1 (SCD1), the Δ9-desaturase that facilitates the transformation of saturated to monounsaturated fatty acids, exhibits higher activity levels [25]. Furthermore, increased levels of monounsaturated fatty acids (MUFAs), specifically oleic acid, are incorporated into triglycerides and cholesterol esters, thereby promoting lipid accumulation within the arterial walls. In addition, these findings are consistent with those of previous studies, which have demonstrated a correlation between SCD1 activity and inflammatory signaling as well as foam cell formation [24,42].

The observed alterations in the ratios of saturated fatty acids (e.g., an increase in C16:0/C18:0, a decrease in C18:0/C20:0, and an increase in C20:0/C22:0) are associated with the dysregulation of the ELOVL enzymes, which are responsible for the extension of long- and very-long-chain fatty acids. Impaired ELOVL6 activity has been demonstrated to affect metabolic balance between palmitate and stearate, thereby contributing to the development of insulin resistance and vascular dysfunction [22]. The disruption of ELOVL1/3 functions has been demonstrated to induce a decline in the synthesis of very-long-chain fatty acids, which are essential for maintaining membrane lipid integrity. Consequently, such an imbalance may lead to complications in endothelial cell stability [23].

The study also demonstrates lower levels of total HUFA in AS patients, which provides support for the hypothesis of a systemic deficit in long-chain PUFA biosynthesis. Thus, this further highlights the role of impaired elongase (ELOVL2, ELOVL5) and desaturase (FADS1) activity in disease pathophysiology.

In patients with AS, the omega-6 pathway demonstrates lower concentrations of C20:3n-6 and C20:4n-6, as well as an imbalance in precursor–product ratios (e.g., a reduction in C18:3n-6/C20:3n-6 and C18:2-n6/C20:4n-6). The present findings could be associated with reduced activity of Δ5-desaturase (FADS1) and Δ6-desaturase (FADS2). In addition, the omega-3 analysis demonstrates a decrease in EPA (C20:5n-3) content, total omega-3 levels, and a reduced EPA/DPA ratio, which could be attributed to impaired desaturase and elongase activity (ELOVL2/5). The reduced production of arachidonic acid (AA), eicosapentaenoic acid (EPA), and docosahexaenoic acid (DHA) leads to a diminished formation of anti-inflammatory eicosanoids and specialized pro-resolving mediators (SPMs) [30,31]. Imbalance in the ratio of n-6 and n-3 metabolism results in increased production of inflammatory lipid mediators, accelerating plaque progression and vessel inflammation.

Arachidonic acid (AA) levels were significantly lower in statin-treated patients compared with healthy controls (*p* = 0.0064), suggesting a potential anti-inflammatory effect. In contrast, AA levels in statin-naive patients did not differ from control samples. The pleiotropic effects of statins on fatty acid metabolism appear to be responsible for the mentioned effect by regulating desaturase expression and inhibiting of inflammatory lipid mediators [43,44].

The hypothesis that plasma arachidonic acid levels decrease in response to statin treatment is based on the premise that this occurs through the regulation of desaturase enzymes (particularly FADS1) or by reducing overall lipid turnover rates [45]. As demonstrated in the published research, statin treatment has reduced the availability of pro-inflammatory omega-6 PUFA substrates, such as AA, thereby decreasing potential eicosanoid production [44].

The trend data for statistical assessment demonstrated that atherosclerosis patients not receiving statins (Statin−) exhibited elevated ratios of arachidonic acid to eicosapentaenoic acid (AA/EPA). However, these did not achieve statistical significance (*p* = 0.08). Increases in the AA/EPA ratio have previously been linked to elevated risk of cardiovascular disease and increased inflammatory activity. Consequently, this suggests possible ongoing signs of inflammation and, therefore, a lasting predisposition towards atherogenesis [2,46,47,48]. It has been demonstrated that in cases where patients are prescribed statin medications, the ratio tends to shift towards levels seen in healthy individuals through the restoration of a certain balance between inflammatory lipid mediators. However, the observed change in the current study population was insufficient to produce statistical differences.

Research has demonstrated that patients suffering from atherosclerosis exhibit alterations in their MUFA composition, which may lead to the formation of mead acid (C20:3n-9) from oleic acid through endogenous synthesis during essential fatty acid deficiency (EFAD) (Figure 2). It has been hypothesized that mead acid may indicate metabolic distress associated with desaturase dysfunction in the context of atherosclerosis [49]. However, direct measurements of mead acid levels in atherosclerosis have not yet been conducted. Further studies should investigate the potential of mead acid as a marker for lipid imbalance within the scope of lipidomic research.

Atherosclerotic plaque lipid content provides essential information for a complete understanding of its biological nature [50]. The analysis demonstrates that unstable plaques display significant alterations in unsaturated fatty acids, characterized by elevated levels of MUFAs and reduced HUFAs. These alterations in unsaturated fatty acid composition support modifications in lipid synthesis and enzymatic processes.

The reduced levels of DHA, along with those of EPA and DPA, detected within unstable atherosclerotic plaques, suggest potential issues with lipid-mediated anti-inflammatory signaling. These long-chain omega-3 polyunsaturated fatty acids (PUFAs) are essential precursors for specialized pro-resolving mediators (SPMs), such as resolvins and protectins. SPMs have multiple crucial functions in inflammation processes, including limiting leukocyte infiltration, blocking vascular smooth muscle cell migration and reducing pro-inflammatory cytokine production [51,52]. Insufficient levels of PUFAs have been shown to negatively impact the resolution of inflammation in the plaque microenvironment, potentially leading to increased plaque instability and a heightened risk of rupture.

Given that previous studies indicate omega-3 fatty acids possess anti-inflammatory properties, future research should incorporate inflammatory markers such as CRP, IL-6, and TNF-α, which may correlate with changes in fatty acids and enhance the understanding of the inflammatory basis of plaque vulnerability [53,54].

Significantly, the measurements from unstable atherosclerotic plaques showed lower levels of arachidonic acid (C20:4n-6) compared with those in stable plaques, despite this compound being essential for the production of pro-inflammatory eicosanoids. The decline of HUFA, when considered in conjunction with this observation, suggests that poor FADS1 activity is required to convert C20:3n-6 into C20:4n-6 [31]. Lower levels of arachidonic acid might result from enzymatic limitations caused by decreased FADS1 gene expression. Alternatively, this decrease could stem from substrate depletion or the regulation of inflammatory signals during advanced plaque development, which is linked to processes of inflammatory resolution.

It is suggested that lowering levels of very-long-chain saturated fatty acids (VLC-SFAs), such as behenic acid (C22:0) and lignoceric acid (C24:0), in unstable atherosclerotic plaques may affect membrane structure and inflammatory signaling. In lipid rafts, which are specialized membrane microdomains, VLC-SFAs have been found to enhance tight lipid packing, which helps maintain membrane rigidity and aids in the spatial organization of receptor clusters that are crucial for effective signal transduction [55,56]. Disruption of these domains can alter the localization and function of the signaling complex toll-like receptor 4 (TLR4), a key mediator of macrophage-driven inflammatory responses in atherosclerosis [57]. It is therefore hypothesized that the depletion of VLC-SFAs may compromise membrane integrity and enhance pro-inflammatory signaling through dysregulated TLR4 activation [58].

The observed alterations in lipid metabolism can be attributed to the upregulation of stearoyl-CoA desaturase-1 (SCD1) and the impairment of FADS1/2 and ELOVL2/5 activity, which in turn give rise to conditions conducive to inflammation, endothelial dysfunction, and plaque instability [52,59,60]. Unstable carotid plaques have been shown to have increased levels of MUFAs and reduced levels of omega-3 HUFAs compared with stable plaques. This was attributed to the fact that fatty acid remodelling may be a metabolic marker for unstable plaques [50]. This finding supports the hypothesis that local and systemic lipid disturbances are mechanistically linked to atherogenesis.

It was also demonstrated that statins modified the relationship between omega-3 fatty acids and lipid markers, affecting atherosclerosis development. In statin-naive patients, omega-3 EPA + DHA levels were strongly associated with improved lipid profiles, including an inverse correlation with triglycerides (R^2^ = 0.453, *p* = 0.047) and a positive correlation with HDL-C (R^2^ = 0.626, *p* = 0.011). These results confirm that omega-3 fats enhance HDL functionality and reduce lipoprotein atherogenicity through established mechanisms [40]. Inverse trends with VLDL-C, LDL-C, and total cholesterol were also observed in the statin-naive group, consistent with the role of omega-3 FAs in suppressing hepatic lipogenesis and enhancing β-oxidation [37,41].

The statistical relationships between omega-3 FAs and lipid markers remained low and insignificant among patients taking statins because statins exert potent effects on LDL cholesterol and triglyceride levels, potentially masking the additional influence of omega-3 FAs [38,39]. The weak TG correlation with an R^2^ = 0.082 and *p* = 0.077 demonstrates that omega-3 FAs still provide additional benefit.

Our data suggest that statins reduce the correlation between EPA + DHA (omega-3 index) and lipid levels, making biomarker interpretation more complex. A plausible explanation is that statins’ strong capabilities in reducing LDL-C and TG may conceal the impact of omega-3 fatty acids on these values. In addition, statins may induce changes in fatty acid metabolism (e.g., by modifying desaturase and elongase enzyme activity or reducing the production of inflammatory lipids) that alter the relationship between omega-3 levels and lipid profile [43,45,52]. In statin-naive patients, omega-3 fatty acids showed their expected beneficial correlations with lipids, whereas under statin therapy these correlations were attenuated by the pharmacological effects of the statins.

The primary factor that distinguished treatment groups during the analysis was statin therapy. Several patients (*n* = 5 out of 12) in the statin-naive group were taking medications that affect lipid metabolism, particularly β-blockers. This study did not analyze this small subgroup separately due to its limited number of participants. The use of β-blockers has been linked to minor changes in lipid profiles, resulting in higher triglyceride levels and lower HDL-C levels. However, these effects depend on β-blocker type and individual patient characteristics [61]. Some studies indicate that β-blockers reduce the ability of omega-3 polyunsaturated fatty acids to lower lipids through possible effects on hepatic lipid metabolism and lipoprotein clearance mechanisms [62,63,64]. The relationship between β-blockers and omega-3 status and function needs additional research in larger study groups because current evidence is insufficient.

The research proceeded by applying stratification methods to compare β-blocker users and non-users within the statin-positive cohort. The lipid markers, including VLDL-C, LDL-C, HDL-C, total cholesterol (TC), and triglycerides (TG), indicated no significant statistical associations in both the statin + β-blocker and statin-only groups. While VLDL-C and triglycerides showed inverse relationships in the statin-only group, these statistical relationships did not achieve significance. The changes in LDL-C and total cholesterol levels remained insignificant regardless of omega-3 fatty acid concentration in both groups. The data revealed weak and non-significant positive trends for HDL-C levels. The slopes and R^2^ values in the statin + β-blocker group were consistently lower or similarly non-significant compared with those in the statin-only group, suggesting that β-blockers neither enhance nor contribute additional lipid-modifying effects in this pharmacologic context. These findings suggest that omega-3 fatty acids exhibit limited lipid-modifying effects, masked by statin pharmacodynamic actions [41], with minimal independent influence from β-blockers.

These results highlight that omega-3 profiles are particularly informative in statin-naive patients and may support personalized lipid management strategies when pharmacological confounders are minimized. The interpretation of biomarkers for cardiovascular risk assessment necessitates separate analysis based on treatment status.

We also developed a mathematical tool to enhance the clinical utility of fatty acid profiling. Although fatty acid analysis is a well-established method in lipidomics and cardiovascular research, there is currently no standardized approach for converting fatty acid values between different blood fractions, such as plasma, whole blood, and erythrocytes. This limits the comparability of results across studies using different sample types. Developing accurate conversion models is therefore a priority. Such models are especially important for analyzing dried blood spots (DBS), which are increasingly being used due to their ease of collection and storage. Mathematical algorithms enable the estimation of erythrocyte fatty acid composition from DBS data, enhancing the consistency and clinical relevance of DBS-based measurements. This is critical for integrating blood plasma and DBS into broader clinical and epidemiological applications and aligning its results with traditional venous blood samples [16,65,66].

The research data show that the logit-based regression method provided better prediction accuracy in converting plasma EPA + DHA values to estimated erythrocyte levels than the Hu et al. (2017) model achieves (R^2^ = 0.782 vs. 0.722) [17]. The logit model demonstrated greater accuracy between estimated and actual erythrocyte values in Bland–Altman analysis due to its provision of narrower limits of agreement and a lower mean bias, thereby enhancing its practical utility for clinical use.

The logit–logistic transformation probably produced these improvements by converting 0–1 proportional data into unbounded linear space. This procedure enables more accurate linear regression modelling when dealing with percentage-based biomarkers, including the omega-3 index, which solves data irregularities caused by raw proportion measurements [16,67].

Population-specific calibration is essential, as the varying model performance results demonstrate its necessity. The Hu et al. (2017) model originates from a worldwide meta-analysis that included results from 56 research projects primarily conducted in Western countries [17]. In contrast, our model was constructed using data from a Russian population (Moscow region), whose dietary patterns can differ from those of North American and Western European populations. Previous worldwide research discovered substantial differences between blood fraction omega-3 fatty acid content across different regions. The research findings validate the need to verify conversion models in local nutritional contexts for specific target populations [68].

External validation in ethnically and geographically diverse cohorts is necessary to assess the generalizability of this model, as fatty acid metabolism is known to vary with dietary patterns and genetic polymorphisms affecting desaturase and elongase activity [16,69].

### Limitations

This study has several limitations. First, the sample size (52 patients, 50 controls) is relatively small, which reduces statistical power, particularly for subgroup analyses involving statin therapy (such as statin-naïve vs. statin-treated) and beta-blocker therapy. Second, the cross-sectional study design does not allow conclusions to be drawn about causality or the longitudinal progression of changes in the fatty acid profile in relation to medication effects or plaque stability. Third, variability in medication types, dosages, and the use of additional drugs introduces potential confounding factors. Fourth, dietary intake, which significantly influences fatty acid composition, was not assessed, limiting the interpretation of metabolic versus dietary influences. Fifth, the developed mathematical model for estimating erythrocyte omega-3 index from plasma fatty acids was calibrated for a specific Russian population, thus requiring validation in other demographic or ethnic groups. Finally, clinical endpoints such as cardiovascular events were not included; future studies are needed to establish the clinical relevance of the observed biochemical and histological associations.

## 5. Conclusions

The present study demonstrates associations between atherosclerosis and notable alterations in the fatty acid composition of blood plasma and plaque, consistent with enzymatic dysregulation of desaturases and elongases. In particular, patients with AS had elevated plasma levels of monounsaturated fatty acids (MUFAs) and lower plasma levels of highly unsaturated fatty acids (HUFAs), including omega-3 polyunsaturated fatty acids (PUFAs) such as EPA and DHA. A similar imbalance was observed in atherosclerotic plaques, with unstable carotid plaques showing increased MUFA levels and reduced omega-3 HUFA levels compared with stable plaques. These findings offer valuable insights into the mechanisms of atherogenesis by linking local and systemic lipid disturbances to the formation of plaques.

In addition to assessment of fatty acid composition, our research also demonstrates that pharmacological treatment with statins modulates the diagnostic value of plasma omega-3 levels. The associations between omega-3 fatty acids and beneficial lipid parameters such as HDL-C and triglycerides remained significant in statin-naïve patients but were attenuated in those receiving statin therapy. Furthermore, the data suggest that β-blockers do not exert additional effects on lipid parameters in statin-treated individuals, indicating that statins primarily mediate the observed changes in lipid profiles. Despite the relatively small sample size, the consistency of our findings with established biological mechanisms lends support to their validity.

To support clinical translation, a logit-based regression model was developed and validated for estimating the erythrocyte omega-3 index from plasma measurements. This model demonstrated strong predictive accuracy and outperformed a previously published Western-population-based model when applied to a Russian cohort, emphasizing the importance of population-specific calibration of the estimation model. This approach enables the integration of fatty acid profiling into broader cardiovascular risk assessment frameworks, especially in settings where red blood cell data are unavailable.

Overall, the findings of this study demonstrate the potential of fatty acid profiling as a diagnostic and therapeutic tool for monitoring carotid atherosclerosis. The assay demonstrates sensitivity to metabolic and inflammatory conditions affecting atherosclerosis. It demonstrates the potential for personalized monitoring of therapeutic responses, including assessment of omega-3 treatment and lipid-lowering drugs and the detection of enzymatic dysregulation. Incorporating fatty acid profiling into clinical practice could facilitate cardiovascular risk stratification by identifying individuals with suboptimal lipidomic patterns who may be at greater risk of plaque instability.

The metabolic and therapeutic insights gained from this study also underscore the importance of further investigation. In particular, the observed modulation of omega-3 efficacy by statins and β-blockers highlights the necessity for personalized strategies in cardiovascular risk management that consider drug–nutrient interactions. It is equally important to recognize that plasma fatty acid profiling may be influenced by comorbid conditions and might not be disease-specific. Future research should assess whether this approach can serve as a primary or complementary biomarker within precision medicine frameworks. Such efforts will be essential for translating fatty acid profiling into improved personalized care for patients with atherosclerosis and for evaluating the omega-3 index as a complementary therapeutic target.

## Figures and Tables

**Table 1:** Clinical and demographic information.

Variables *n* (%)	Group 1—Control *n* = 50	Group 2—AS *n* = 52	*p*-Value
Overall (Total *n* = 102)	50 (49.0%)	52 (51.0%)	>0.9999
**Demographic**			
Age, year (Mean ± SD)	46 ± 11.6	67 ± 8.4	<0.0001
Obese (BMI ≥ 30 kg/m^2^)	9 (18.0%)	14 (26.9%)	0.3462
Male	28 (56.0%)	40 (76.9%)	0.0353
Female	22 (44.0%)	12 (23.1%)	0.0353
**Comorbidities**			
Smokers	6 (12.0%)	25 (48.1%)	<0.0001
DM	2 (4.0%)	12 (23.1%)	0.0078
HTN	12 (24.0%)	47 (90.4%)	<0.0001
IHD	0 (0.0%)	18 (34.6%)	<0.0001
IS	0 (0.0%)	16 (30.8%)	<0.0001
HFrEF	0 (0.0%)	4 (7.7%)	0.1179
Paroxysmal atrial fibrillation	0 (0.0%)	1 (1.9%)	>0.9999
CKD	3 (6.0%)	27 (51.9%)	<0.0001
**Medication Use**			
Statin therapy	0 (0.0%)	40 (76.9%)	<0.0001
Statin therapy + BB (out of statin therapy group *n* = 40)	-	25 (62.5%)	-
Thiazide diuretic	0 (0.0%)	3 (5.8%)	0.2429
Loop diuretic	0 (0.0%)	2 (3.8%)	0.4952
Indapamide (thiazide-like diuretic)	1 (2.0%)	6 (11.5%)	0.1125
Xanthine oxidase inhibitors	0 (0.0%)	5 (9.6%)	0.0566

BMI = body mass index; DM = diabetes mellitus; HTN = hypertension; IHD = ischemic heart disease; IS = ischemic stroke; HFrEF = heart failure with left ventricular ejection fraction less than 40%; CKD = chronic kidney disease; BB = β-blocker.

**Table 2:** Concentrations of broad fatty acids classes and selected fatty acids in plasma and plaques.

Parameter, %	Plasma		Plaques	
	Control (*n* = 50)	AS (*n* = 52)	Stable (*n* = 28)	Unstable (*n* = 24)
**Broad FA classes and omega-3 index**				
SFA	37.61 ± 3.88 ***	34.04 ± 1.38	33.97 ± 6.40	31.66 ± 5.16
UFA	62.40 ± 3.88 ***	65.96 ± 1.38	66.03 ± 6.40	68.34 ± 5.16
MUFA	26.15 ± 3.64 ***	30.94 ± 3.50	30.90 ± 4.15 **^#^**	33.62 ± 5.11
PUFA	35.77 ± 4.47	34.80 ± 3.11	36.04 [30.68–38.66]	34.68 [32.80–35.95]
HUFA	12.55 [10.12–14.52] ***	10.31 [9.25–11.78]	11.91 ± 1.79 **^#^**	10.68 ± 1.74
omega-6	32.68 ± 4.07	32.12 ± 3.13	32.72 ± 4.03	32.31 ± 2.63
EPA + DHA	2.70 ± 1.07 **	2.23 ± 0.58	1.90 [1.54–2.48] **^#^**	1.76 [1.34–1.90]
omega-3 index	4.92 ± 1.29 **	4.35 ± 0.72	-	-
TFA	0.40 [0.28–0.69] ***	0.31 [0.16–0.47]	0.33 ± 0.13	0.35 ± 0.15
**Selected FA: SFA, MUFA pathway**				
C16:0	26.60 [24.70–27.91] ***	25.12 [24.17–26.00]	23.07 ± 3.23	22.03 ± 3.15
C18:0	9.27 ± 1.83 ***	7.48 ± 0.97	7.59 [5.99–10.18]	6.92 [5.87–8.24]
C20:0	0.19 [0.13–0.23]	0.19 [0.17–0.20]	0.14 ± 0.05 **^#^**	0.11 ± 0.04
C22:0	0.43 [0.33–0.56] ***	0.36 [0.32–0.42]	0.41 ± 0.13 **^#^**	0.35 ± 0.08
C24:0	0.33 [0.28–0.44] ***	0.28 [0.24–0.33]	0.54 ± 0.16 **^#^**	0.46 ± 0.14
C16:1n-7	2.19 [1.71–2.76]	2.12 [1.74–2.81]	2.79 [2.16–3.33] **^#^**	3.02 [2.56–3.79]
C18:1n-9	23.14 ± 3.38 ***	27.92 ± 3.24	26.86 ± 3.62 **^#^**	29.36 ± 4.31
C20:1n-9	0.14 [0.12–0.18] ***	0.17 [0.16–0.20]	0.26 ± 0.11 **^#^**	0.33 ± 0.15
**Selected FA: omega-6, omega-3 pathway**				
C20:3n-6	1.31 [1.10–1.54] *	1.14 [0.94–1.45]	1.69 [1.06–1.99]	1.50 [1.12–1.95]
C20:4n-6	8.06 [6.25–9.01] **	6.29 [5.48–7.10]	7.02 ± 0.97 **^##^**	6.17 ± 1.23
DPA, C22:5n-3	0.37 [0.31–0.45]	0.34 [0.30–0.38]	0.38 [0.27–0.47] **^#^**	0.26 [0.23–0.36]
EPA, C20:5n-3	0.56 [0.34–0.79] **	0.42 [0.30–0.56]	0.57 [0.37–0.78]	0.48 [0.34–0.62]
DHA, C22:6n-3	2.06 [1.34–2.55]	1.80 [1.40–2.06]	1.29 [1.16–1.62] **^#^**	1.11 [0.96–1.35]

Note: Chosen parameters differ significantly: *, **, ***—*p* < 0.05, *p* < 0.01, *p* < 0.001 accordingly when comparison control plasma to AS group plasma; ^#^, ^##^—*p* < 0.05, *p* < 0.01 accordingly when comparison in stable plaques to unstable ones. For normally distributed values, the values are given in Mean ± SD format, for values with non-normal distribution Median [Q1; Q3]. The complete list of analysed parameters and exact *p*-values is presented in File S1, Tables S2 and S3.

## Data Availability

The data can be provided at the official request of the Principal Investigator because our local ethics committee does not allow them to be provided openly.

## Supplementary Materials

The following supporting information can be downloaded at https://www.mdpi.com/article/10.3390/biomedicines13061383/s1, Table S1: General information about the sample to develop a mathematical model for predicting omega-3 index in erythrocytes from plasma data; Table S2: Blood plasma FA concertation comparison, broad FA classes, standard indexes, metabolic balance ratios; Table S3: Atherosclerotic plaques FA concertation comparison, broad FA classes, standard indexes, metabolic balance ratios; Table S4: Bland–Altman analysis for three models (CI stands for confidence interval, LOA for the limit of agreement), all values, except *p*-value, in %; Figure S1: Representative histological features of stable (1–3) and unstable (4–6) atherosclerotic plaques; Figure S2: Q–Q plots of regression residuals before (left) and after (right) logit transformation of the Omega-3 index. The logit-transformed model demonstrates improved normality, with residuals more closely aligned to the theoretical distribution. The solid line represents the theoretical quantiles of a normal distribution, while the gray shaded area indicates the 95% confidence envelope for expected sampling variability; Figure S3: Comparison between the Hu et al. (2017) mode (Hu2017) [17] and observed data (left) and logit model (right). Light gray area represents 95% confidence bands (α = 0.05); Figure S4: Bland–Altman plots for three models under consideration: linear predictor for Omega-3 index (left); linear predictor for logit transformed data (center); linear predictor due to Hu et al. (2017) model [17] (right); Figure S5: The correlation between omega-3 fatty acid levels and lipid markers VLDL-C, LDL-C, HDL-C, total cholesterol and triglycerides in statin-treated (Statin+, *n* = 40) and statin-naïve (Statin−, *n* = 12) patients.; Figure S6: The correlation between omega-3 fatty acid levels and lipid markers VLDL-C, LDL-C, HDL-C, total cholesterol, and triglycerides considering β-blockers intake, statin + β-blocker (Statin + BB, *n* = 25) and statin-only (Statin only, *n* = 15) groups.
