# Supplementary material for: Plasma Fatty Acid Profiling and Mathematical Estimation of the Omega-3 Index: Toward Diagnostic Tools in Atherosclerosis and Statin Therapy Monitoring
Source: Biomedicines. 2025 Jun 4;13(6):1383. doi: 10.3390/biomedicines13061383 (PMC12189591; doi:10.3390/biomedicines13061383)
Supplement: Supplementary file 1 [file biomedicines-13-01383-s001.zip › biomedicines-3632980-supplementary.pdf]

Supplementary material to the Article

Plasma Fatty Acid Profiling and Mathematical Estimation of the Omega-3 Index: Toward Diagnostic Tools in Atherosclerosis and Statin Therapy Monitoring

Supplemental Information

Table S1. General information about the sample to develop a mathematical model for predicting omega-3 index in erythrocytes from plasma data.

Selection	Count	Mean Age	Mean Omega-3 index, %	Median Omega-3 index, %	Omega-3 standard deviation, %
Male	29	49	5.12	5.10	1.71
Female	40	43	5.17	5.14	1.34
Age < 36	18	28	5.22	5.00	1.82
36 ≤ Age < 47	18	41	5.06	5.44	1.55
47 ≤ Age < 58	16	52	5.25	5.39	0.96
58 ≤ Age	17	63	5.06	4.97	1.59
Healthy	50	42	5.30	5.32	1.57
Diseases	19	56	4.73	4.55	1.21

Table S2. Blood plasma FA concertation comparison, broad FA classes, standard indexes, metabolic balance ratios.

Variables	Group 1 Control n = 50	Group 2 AS n = 52	*p*-Value
**Broad FA classes and omega-3 levels**			
SFA, %, Mean ± SD	37.61 ± 3.88	34.04 ± 1.38	<0.0001
UFA, %, Mean ± SD	62.40 ± 3.88	65.96 ± 1.38	<0.0001
MUFA, %, Mean ± SD	26.15 ± 3.64	30.94 ± 3.50	<0.0001
PUFA, %, Mean ± SD	35.77 ± 4.47	34.80 ± 3.11	0.2066
HUFA, %, Median [IQR25%–75%]	12.55 [10.12–14.52]	10.31 [9.25–11.78]	0.0009
omega-6, %, Mean ± SD	32.68 ± 4.07	32.12 ± 3.13	0.4405
omega-3 EPA+DHA, %, Mean ± SD	2.70 ± 1.07	2.23 ± 0.58	0.0068
TFA, %, Median [IQR25%–75%]	0.40 [0.28–0.69]	0.31 [0.16–0.47]	<0.0001
**SFA, MUFA pathway**			
**Selected FA**			
C16:0, Median [IQR25%–75%]	26.60 [24.70–27.91]	25.12 [24.17–26.00]	0.0007
C18:0, %, Mean ± SD	9.27 ± 1.83	7.48 ± 0.97	<0.0001
C20:0, %, Median [IQR25%–75%]	0.19 [0.13–0.23]	0.19 [0.17–0.20]	0.6083
C22:0, %, Median [IQR25%–75%]	0.43 [0.33–0.56]	0.36 [0.32–0.42]	0.0047
C24:0, %, Median [IQR25%–75%]	0.33 [0.28–0.44]	0.28 [0.24–0.33]	0.0009
C18:1 n-9, %, Mean ± SD	23.14 ± 3.38	27.92 ± 3.24	<0.0001
C20:1n-9, %, Median [IQR25%–75%]	0.14 [0.12–0.18]	0.17 [0.16–0.20]	<0.0001
C24:1n-9, %, Median [IQR25%–75%]	0.51 [0.39–0.77]	0.56 [0.51–0.64]	0.7668
C16:1n-7, Median [IQR25%–75%]	2.19 [1.71–2.76]	2.12 [1.74–2.81]	0.8559
**Metabolic pathway equilibrium ratios**			

C16/C16:1n-7, Median [IQR25%–75%]	11.88 [10.08–15.89]	11.92 [8.75–14.16]	0.5341
C16:0/C18:0, Median [IQR25%–75%]	2.78 [2.49–3.36]	3.34 [3.06–3.69]	<0.0001
C18:1n-9/C20:1n-9, Median [IQR25%–75%]	171.20 [131.20–187.60]	160.80 [145.50–174.70]	0.4914
C20:1n-9/C24:1n-9, Median [IQR25%–75%]	0.27 [0.17–0.36]	0.28 [0.25–0.35]	0.1953
C18/C18:1n-9, Median [IQR25%–75%]	0.40 [0.29–0.53]	0.27 [0.23–0.31]	<0.0001
C18:0/C20:0, Median [IQR25%–75%]	49.73 [41.38–58.22]	39.77 [36.33–42.55]	<0.0001
C20:0/C22:0, Median [IQR25%–75%]	0.40 [0.34–0.46]	0.52 [0.48–0.58]	<0.0001
C22:0/C24:0, Median [IQR25%–75%]	1.22 [1.14–1.33]	1.27 [1.23–1.34]	0.0348
**Omega-3, -6 pathway**			
**Selected FA**			
C18:2 n-6, %, Mean ± SD	22.45 ± 4.74	22.95 ± 3.26	0.5324
C18:3n-6, %, Median [IQR25%–75%]	0.81 [0.69–1.03]	0.98 [0.74–1.23]	0.0748
C20:3 n-6, %, Median [IQR25%–75%]	1.31 [1.10–1.54]	1.14 [0.94–1.45]	0.0303
C20:4 n-6, %, Median [IQR25%–75%]	8.06 [6.25–9.01]	6.29 [5.48–7.10]	0.0033
C22:4 n-6, Median [IQR25%–75%]	0.16 [0.13–0.21]	0.17 [0.14–0.21]	0.2977
DPA, C22:5 n-3, %, Median [IQR25%–75%]	0.37 [0.31–0.45]	0.34 [0.30–0.38]	0.1115
EPA, C20:5 n-3, %, Median [IQR25%–75%]	0.56 [0.34–0.79]	0.42 [0.30–0.56]	0.0159
DHA, C22:6 n-3, %, Median [IQR25%–75%]	2.06 [1.34–2.55]	1.80 [1.40–2.06]	0.0692
**Ratio-based index**			
AA/EPA, Median [IQR25%–75%]	12.98 [7.47–18.45]	15.87 [11.45–20.16]	0.0715
AA/DPA, Median [IQR25%–75%]	20.57 [15.67–24.73]	19.47 [14.75–22.40]	0.2423
AA/DHA, Median [IQR25%–75%]	3.77 [3.07–4.33]	3.40 [2.92–4.58]	0.6225
AA/EPA+DHA, Median [IQR25%–75%]	2.87 [2.16–3.51]	2.91 [2.27–3.96]	0.6083
**Metabolic pathway equilibrium ratios**			
C18:2n-6/C18:3n-6, Median [IQR25%–75%]	25.07 [18.87–38.00]	22.15 [17.06–34.22]	0.1718
C18:3n-6/C20:3n-6, Median [IQR25%–75%]	1.50 [1.17–2.09]	0.84 [0.62–1.07]	<0.0001
C20:3n-6/C20:4n-6, Median [IQR25%–75%]	0.18 [0.14–0.22]	0.18 [0.14–0.24]	0.8769
C20:4n-6/C22:4n-6, Median [IQR25%–75%]	42.27 [36.56–50.05]	36.54 [31.24–43.90]	0.0006
EPA/DPA, Median [IQR25%–75%]	1.41 [1.04–1.84]	1.21 [0.98–1.49]	0.0371
DPA/DHA, Median [IQR25%–75%]	0.20 [0.17–0.23]	0.19 [0.17–0.23]	0.7566
DHA/DPA, Median [IQR25%–75%]	5.08 [4.35–5.76]	5.14 [4.37–5.91]	0.9535
C18:2n-6/C20:3n-6, Median [IQR25%–75%]	17.43 [12.44–23.49]	19.47 [14.98–26.50]	0.1226
C18:2n-6/C20:4n-6, Median [IQR25%–75%]	2,69 [2,126–3,584]	3,634 [3,014–4,513]	0.0054

Table S3. Atherosclerotic plaques FA concertation comparison, broad FA classes, standard indexes, metabolic balance ratios.

Variables	Group 1 Stable n = 28	Group 2 Unstable n = 24	*p*-Value
**Broad FA classes and omega-3 levels**			
SFA, %, Mean ± SD	33.97 ± 6.40	31.66 ± 5.16	0.1473
UFA, %, Mean ± SD	66.03 ± 6.40	68.34 ± 5.16	0.1473
MUFA, %, Mean ± SD	30.90 ± 4.15	33.62 ± 5.11	0.0323
PUFA, %, Mean ± SD	36.04 [30.68–38.66]	34.68 [32.80–35.95]	0.5543
HUFA, %, Median [IQR25%–75%]	11.91 ± 1.79	10.68 ± 1.74	0.0129
omega-6, %, Mean ± SD	32.72 ± 4.03	32.31 ± 2.63	0.6559
omega-3 EPA+DHA, %, Mean ± SD	1.90 [1.54–2.48]	1.76 [1.345–1.895]	0.0389
TFA, %, Median [IQR25%–75%]	0.33 ± 0.13	0.35 ± 0.15	0.5135
**SFA, MUFA pathway**			
**Selected FA**			
C16:1n-7, %, Median [IQR25%–75%]	2.79 [2.16–3.34]	3.02 [2.56–3.79]	0.0361
C18:1 n-9, %, Mean ± SD	26.86 ± 3.62	29.36 ± 4.31	0.0217

C20:1n-9, %, Mean ± SD	0.26 ± 0.11	0.33 ± 0.15	0.0407
C24:1n-9, %, Median [IQR25%–75%]	0.84 [0.71–1.22]	0.96 [0.75–1.11]	0.9448
C16:0, %, Mean ± SD	23.07 ± 3.23	22.03 ± 3.15	0.2285
C18:0, %, Median [IQR25%–75%]	7.59 [5.99–10.18]	6.92 [5.87–8.24]	0.2939
C20:0, %, Mean ± SD	0.14 ± 0.05	0.11 ± 0.04	0.0303
C22:0, %, Mean ± SD	0.41 ± 0.13	0.34 ± 0.08	0.0238
C24:0, %, Mean ± SD	0.54 ± 0.16	0.46 ± 0.14	0.0410
**Omega-3, -6 pathway**			
**Selected FA**			
C18:2 n-6, %, Mean ± SD	21.99 ± 4.57	22.60 ± 2.37	0.5494
C18:3n-6, %, Median [IQR25%–75%]	0.55 ± 0.18	0.55 ± 0.15	0.9326
C20:3 n-6, %, Median [IQR25%–75%]	1.69 [1.06–2.00]	1.50 [1.12–1.95]	0.6776
C20:4 n-6, %, Mean ± SD	7.02 ± 0.97	6.17 ± 1.23	0.0061
C22:4 n-6, Median [IQR25%–75%]	0.71 [0.55–1.06]	0.66 [0.42–0.97]	0.2671
DPA, C22:5 n-3, Median [IQR25%–75%]	0.38 [0.27–0.47]	0.26 [0.23–0.36]	0.0335
EPA, C20:5 n-3, Median [IQR25%–75%]	0.57 [0.37–0.78]	0.48 [0.34–0.62]	0.1318
DHA, C22:6 n-3, Median [IQR25%–75%]	1.29 [1.16–1.62]	1.11 [0.96–1.35]	0.0247

Table S4. Bland–Altman analysis for three models (CI stands for confidence interval, LOA for the limit of agreement). All values, except *p*-value, are in %.

Parameter	Linear	Logit	Hu2017
p-value (0-hypothesis: bias = 0)	1.00	0.60	0.64
Maximum value for average measures	9.11	8.91	9.27
Minimum value for average measures	2.31	1.96	1.53
Maximum value for difference in measures	1.64	1.65	2.32
Minimum value for difference in measures	−1.63	−1.65	−2.42
Bias	0.00	0.05	−0.06
Standard deviation of bias	0.74	0.72	0.98
Standard error of bias	0.09	0.09	0.12
Standard error for limits of agreement	0.15	0.15	0.20
Bias- upper 95% CI	0.18	0.22	0.18
Bias- lower 95% CI	−0.18	−0.13	−0.29
Upper D	1.45	1.46	1.86
Upper LOA- upper 95% CI	1.76	1.76	2.27
Upper LOA- lower 95% CI	1.14	1.16	1.46
Lower limit of agreement	−1.45	−1.37	−1.98
Lower LOA- upper 95% CI	−1.14	−1.07	−1.57
Lower LOA- lower 95% CI	−1.76	−1.67	−2.38
Mean of differences/means:	−1.52	−0.05	1.61
Spread of data between lower and upper LOAs:	2.90	2.83	3.84

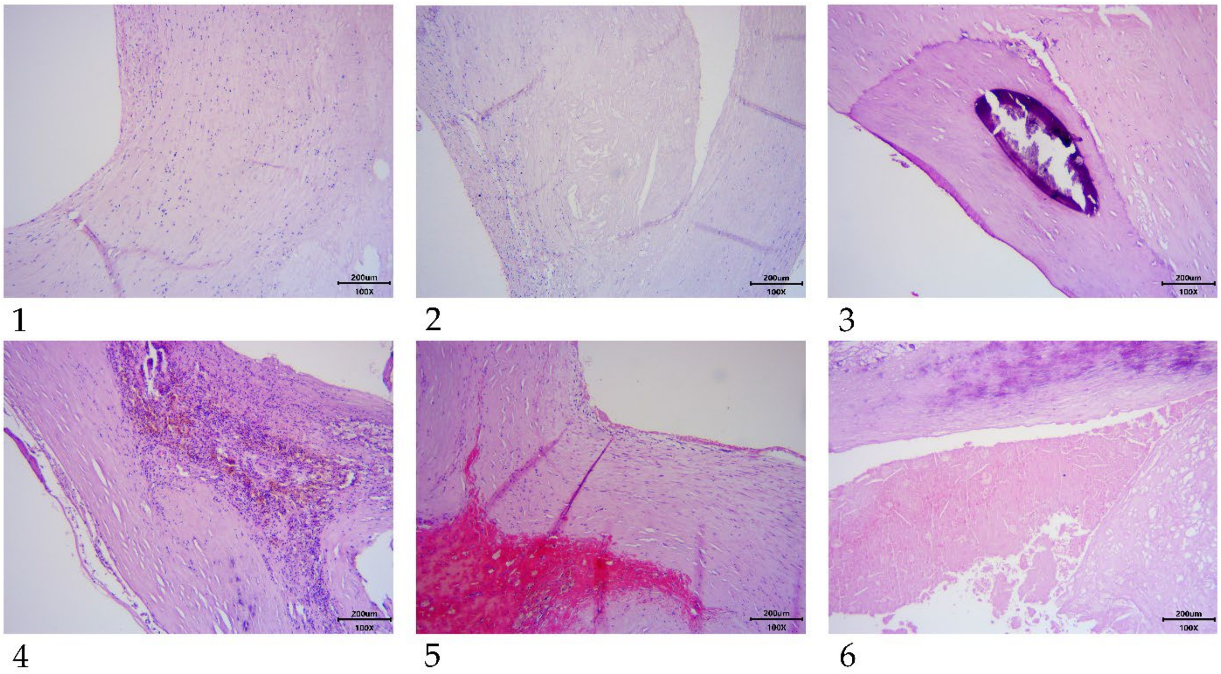

Figure S1. Representative histological features of stable (1–3) and unstable (4–6) atherosclerotic plaques. Hematoxylin and eosin staining, 100× magnification.

1 - Stable plaque with thick fibrous cap (score 3).

2 - Stable plaque with a lipid core with low-grade inflammation (score 1) and neoangiogenesis (score 1).

3 - Stable plaque with a thick fibrous cap (score 3) and focal microcalcifications (score 3).

4 - Unstable plaque with intense inflammatory infiltration (score 3) and neoangiogenesis (score 3).

5 - Unstable plaque exhibiting intraplaque hemorrhage (score 3).

6 - Unstable plaque with atheromatous degeneration, characterized by a large lipid core (score 3) and focal microcalcifications (score 1).

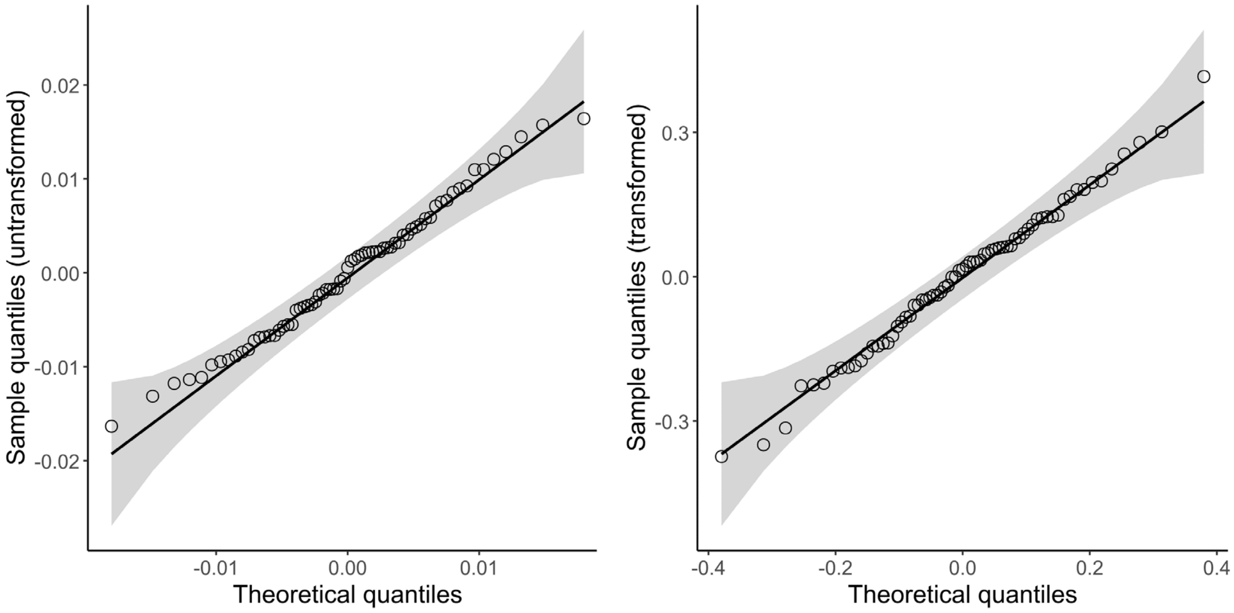

Figure S2. Q–Q plots of regression residuals before (**left**) and after (**right**) logit transformation of the Omega-3 index. The logit-transformed model demonstrates improved normality, with residuals more closely aligned to the theoretical distribution. The solid line represents the theoretical quantiles of a normal distribution, while the grey shaded area indicates the 95% confidence envelope for expected sampling variability.

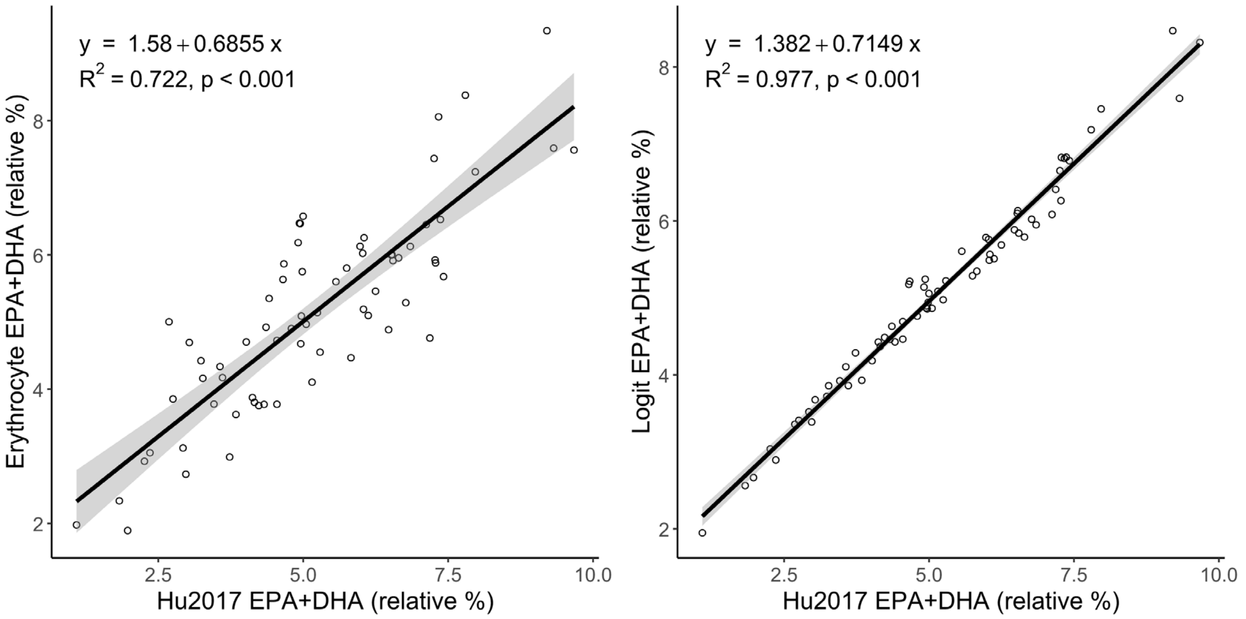

Figure S3. Comparison between the Hu et al. (2017) mode (Hu2017) [17] and observed data (**left**) and logit model (**right**). Light grey area represents 95% confidence bands ($\alpha = 0.05$).

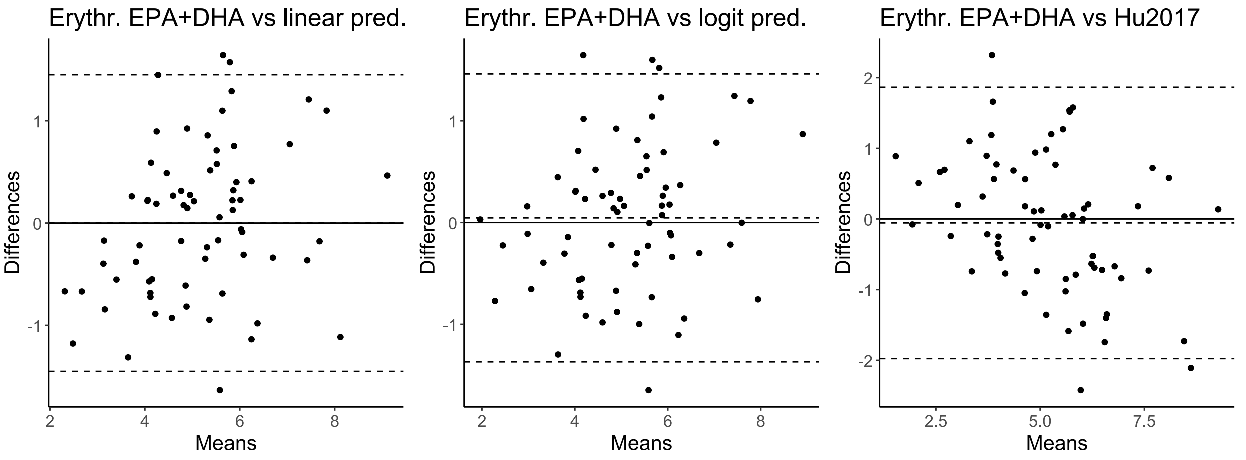

Figure S4. Bland–Altman plots for three models under consideration: linear predictor for Omega-3 index (**left**), linear predictor for logit transformed data (center), linear predictor due to Hu et al. (2017) model [17] (**right**).

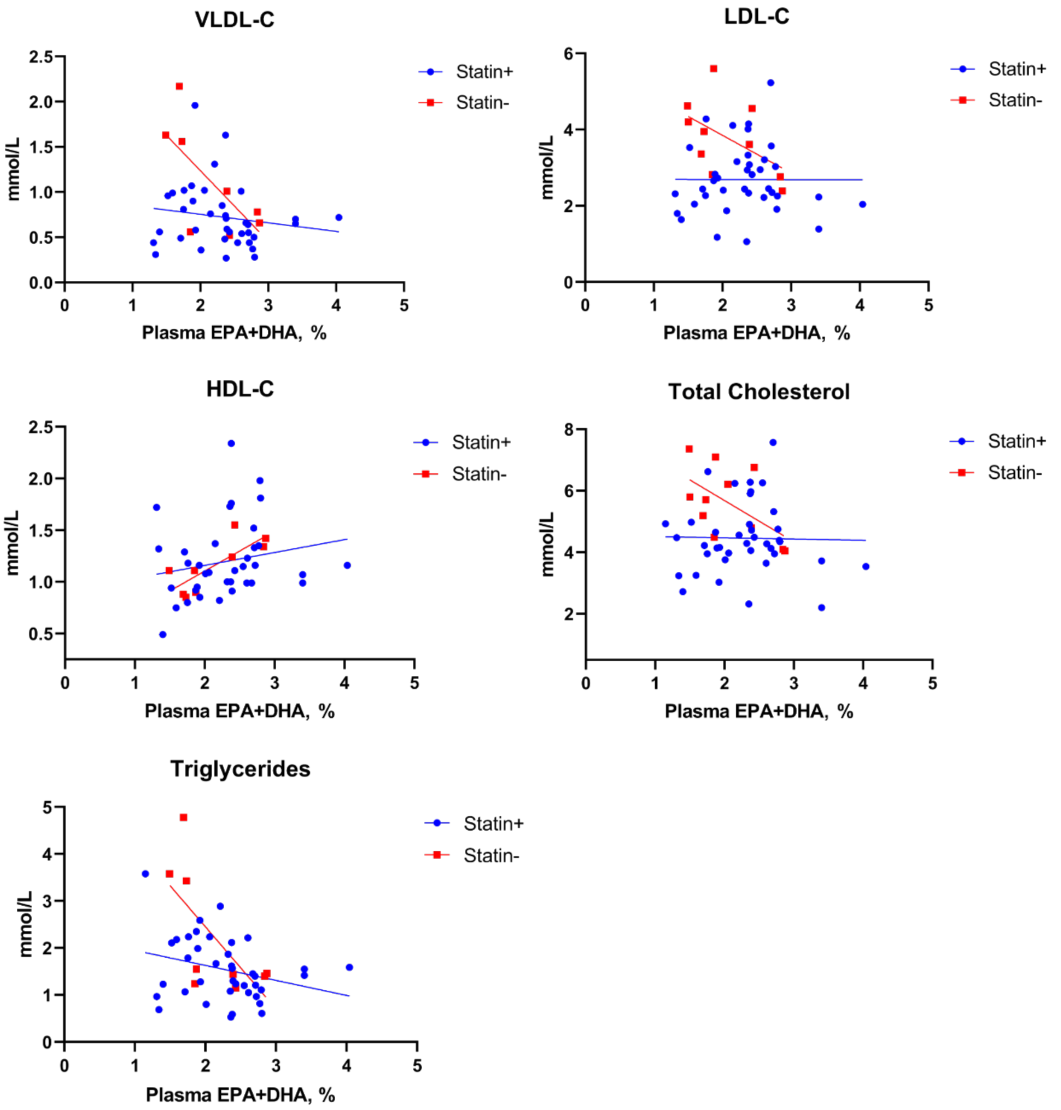

Figure S5. The correlation between omega-3 fatty acid levels and lipid markers VLDL-C, LDL-C, HDL-C, total cholesterol and triglycerides in statin-treated patients (Statin+, n = 40) and statin-naïve (Statin−, n = 12).
VLDL-C: Statin+ $R^2 = 0.024$, $p = 0.357$ vs Statin− $R^2 = 0.485$, $p = 0.055$;
LDL-C: Statin+ $R^2 < 0.001$, $p = 0.989$ vs Statin− $R^2 = 0.271$, $p = 0.123$;
HDL-C: Statin+ $R^2 = 0.035$, $p = 0.265$ vs Statin− $R^2 = 0.626$, $p = 0.011$;
total cholesterol: Statin+ $R^2 = 0.0004$, $p = 0.902$ vs Statin− $R^2 = 0.322$, $p = 0.069$;
triglycerides: Statin+ $R^2 = 0.082$, $p = 0.077$ vs Statin− $R^2 = 0.453$, $p = 0.047$.

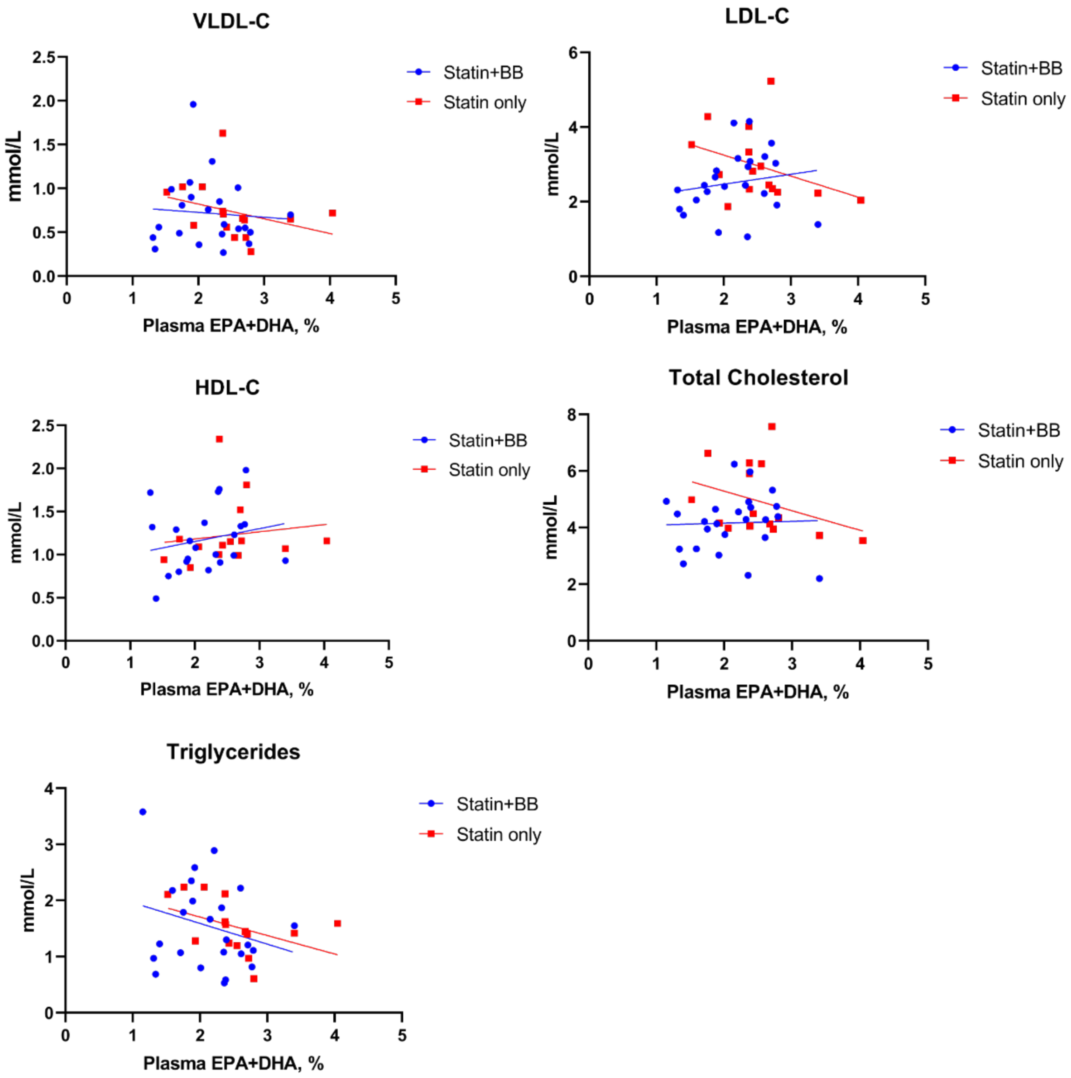

Figure S6. The correlation between omega-3 fatty acid levels and lipid markers VLDL-C, LDL-C, HDL-C, total cholesterol and triglycerides considering β-blockers intake, forming statin + β-blocker (Statin + BB, n = 25) and statin-only (Statin only, n = 15) groups.

VLDL-C: Statin+BB $R^2 = 0.006$, $p = 0.736$ vs Statin only $R^2 = 0.105$, $p = 0.240$;

LDL-C: Statin+BB $R^2 = 0.028$, $p = 0.443$ vs Statin only $R^2 = 0.137$, $p = 0.174$;

HDL-C: Statin+BB $R^2 = 0.045$, $p = 0.341$ vs Statin only $R^2 = 0.018$, $p = 0.635$;

total cholesterol: Statin+BB $R^2 < 0.002$, $p = 0.869$ vs Statin only $R^2 = 0.117$, $p = 0.211$;

triglycerides: Statin+BB $R^2 = 0.069$, $p = 0.217$ vs Statin only $R^2 = 0.188$, $p = 0.107$.
